# Discovery of spontaneous mesoscopic strain waves in nematic domains using dark-field x-ray microscopy

**DOI:** 10.1126/sciadv.aec8998

**Published:** 2026-05-20

**Authors:** Kaan A. Yay, William J. Meese, Elliot Kisiel, Matthew J. Krogstad, Anisha G. Singh, Rafael M. Fernandes, Zahir Islam, Ian R. Fisher

**Affiliations:** ^1^ Geballe Laboratory for Advanced Materials, Stanford University, Stanford, CA, USA.; ^2^ Stanford Institute for Materials and Energy Sciences, SLAC National Accelerator Laboratory, Menlo Park, CA, USA.; ^3^ Department of Physics, Stanford University, Stanford, CA, USA.; ^4^ Department of Physics, The Grainger College of Engineering, University of Illinois Urbana-Champaign, Urbana, IL, USA.; ^5^ Anthony J. Leggett Institute for Condensed Matter Theory, The Grainger College of Engineering, University of Illinois Urbana-Champaign, Urbana, IL, USA.; ^6^ X-ray Science Division, Advanced Photon Source, Argonne National Laboratory, Argonne, IL, USA.; ^7^ Department of Applied Physics, Stanford University, Stanford, CA, USA.

## Abstract

Electronic nematic order arises when correlated electrons spontaneously break the rotational symmetry of a crystal lattice. When electronic nematic order couples bilinearly to symmetry-breaking lattice strain, both appear together at a single ferroelastic phase transition, producing structural twin domains with distinct orientations of the nematic director. While the effects of applied strain on these domains are well established, the intrinsic behavior of spontaneous subdomain strain fields has remained unexplored. Here, we report the discovery of spontaneous mesoscopic strain waves within individual nematic domains of an iron-based superconductor, observed using dark-field x-ray microscopy (DFXM). Using this advanced full-field imaging technique, we visualize subdomain strain modulations emerging concurrently with nematic order. Elastic compatibility relations governing inhomogeneous strains provide a natural mechanism for the strain waves. Our findings reveal a broadly relevant form of strain self-organization and position DFXM as a powerful probe of the local interplay between lattice strain and electronic order.

## Teaser

Advanced x-ray microscopy reveals hidden strain ripples born from electronic nematic order in a quantum material.

## INTRODUCTION

The spontaneous emergence of symmetry-breaking strain is a hallmark of ferroelastic phase transitions in solids (*1*). For temperatures below the critical temperature, the crystal lattice can deform in at least two (depending on the crystal symmetry) equivalent but distinct orientations related to each other by the broken symmetry. To reduce the overall strain energy, the crystal often forms structural twin domains in the broken-symmetry phase: mesoscale regions with differing orientations of the spontaneous lattice deformation (*2*). These domains are then separated by well-defined interfaces called domain walls. While the symmetry properties of structural domains are well established (*2*, *3*), how internal local strain fields behave at the mesoscopic scale inside bulk domains is largely unexplored, especially in quantum materials for which the structural transition is driven by electronic degrees of freedom [e.g., in electronic nematic systems; (*4*–*6*)]. In addition to the spontaneous strain of the broken-symmetry phase, extrinsic strain fields readily form around common lattice imperfections such as dislocations (*7*) and internal boundaries such as domain walls (*8*), where they can reach a magnitude of $5\times 10^{-4}$ and extend for several microns (*9*). Long-range inhomogeneous strain fields at these magnitudes can affect the local electronic properties of a variety of bulk quantum materials. Notably, anisotropic strains can lead to strong resistive anisotropies (*10*, *11*), suppress (*12*) or enhance (*13*) superconductivity, reorder the charge density wave vector (*14*), and drive the band structure across a topological transition (*15*, *16*) for several distinct strongly correlated and topological systems. Thus, it is of critical importance to obtain a quantitative understanding of the local strain variations that must occur at/near domain boundaries. The present study focuses on nematic domains, but the ideas that we advance are more broadly relevant, applying to any system for which spontaneous domain formation results in local strain gradients.

Methods commonly used in condensed-matter physics research to measure strain, however, either lack the spatial resolution to detect strain variation at the micrometer scale or are unable to sample a large enough volume within a bulk crystal. On the one hand, methods such as strain gauges (*11*, *17*), displacement sensors (*18*), and conventional x-ray diffraction (XRD) (*19*, *20*) only give an average picture of the bulk strain at the millimeter scale. On the other hand, high–spatial resolution methods such as transmission electron microscopy require sample thicknesses below 100 nm and are therefore unable to probe strain fields within bulk samples (*2*). Recent application of focused ion beam milling on strongly correlated metals has enabled researchers to tailor the geometry of samples and thus impose specific mechanical boundary conditions at micrometer length scales (*21*, *22*). Achieved strain magnitudes and orientations in the sample are then predicted by finite-element analysis (FEA) simulations. Although the predicted strain values have been in accordance with transport and magnetization measurements in ultraclean samples (*21*), FEA simulations do not take into account local chemical strain due to stoichiometric deviations (*23*) or internal structure such as ferroelastic domain walls, both of which are common in materials of high interest, including doped YBa_2_Cu_3_O_7_ (*2*, *24*) and BaFe_2_As_2_ (*25*, *26*). Thus, we lack mesoscopic studies of internal strain fields in quantum materials.

To address this challenge, we use dark-field x-ray microscopy (DFXM), a full-field imaging technique (*27*). As in dark-field electron microscopy, the idea of DFXM is to insert an objective lens into the path of a particular Bragg peak to obtain a magnified real-space image of the regions of the crystal that are diffracting toward that Bragg peak. Using x-rays instead of electrons, however, enables DFXM to nondestructively image the interior regions of samples of thickness exceeding 100 μm. By positioning the lens downstream of the sample and thanks to the high spatial and angular resolution of modern x-ray lenses (*28*), one can resolve strains of ~10^−5^ in a field of view of 30 to 100 μm with a spatial resolution of 50 to 100 nm. DFXM has been applied successfully to visualize the orientation and strain variations due to crystal heterogeneities in a variety of industrial and functional materials such as aluminum (*27*, *29*) and polycrystalline BaTiO_3_ (*9*). More recently, the extension of DFXM techniques to cryogenic temperatures (*30*) has been used to image the spatial separation of two distinct charge-density wave orders and the related structural twinning in the kagome superconductor CsV_3_Sb_5_ (*31*).

Here, we report the direct observation of coherent micron-scale strain waves within single nematic/ferroelastic domains in the low-temperature orthorhombic phase of the correlated iron pnictide Ba(Fe_0.98_Cu_0.02_)_2_As_2_ using cryogenic DFXM. The strain waves have an amplitude of 10^−5^ with a wavelength of ~2 to 3 μm and run parallel to the orthorhombic twin domain walls. As the twin domain walls disappear in the high-temperature tetragonal phase, so do the strain waves. We propose that the coherent strain waves emerge to satisfy the local elasticity compatibility relations of inhomogeneous strain fields and the mechanical boundary conditions imposed on the domain by its boundaries. Our result highlights that strain must be treated as a spatially varying local field rather than a uniform field because mesoscopic strain variations must obey additional geometrical constraints.

## RESULTS

### Nematic domains in iron pnictides

Ba(Fe_1−*x*_Cu*_x_*)_2_As_2_ is an iron pnictide in the same family as the archetypal iron–based superconductor Ba(Fe_1−*x*_Co*_x_*)_2_As_2_ (*32*–*34*). The parent compound BaFe_2_As_2_ undergoes two phase transitions near 134 K: first, a ferroelastic structural phase transition at $T_{S}$, and, then, an antiferromagnetic transition at $T_{N}$. Similar to Co doping of the Fe sites, Cu doping suppresses both of these transitions to lower temperatures (*35*). In contrast to the extensive superconducting dome induced by Co doping, however, Cu doping is reported to induce superconductivity only in the vicinity of a doping level of x=4.4% with an optimal critical temperature $\left(T_{c}\right)=2.1$ K (*35*), presumably due to the pair-breaking effects of Cu (*36*). The sample that we used in our measurements was a single crystal of Ba(Fe_0.98_Cu_0.02_)_2_As_2_ (Cu-Ba122) with two well-defined phase transitions at $T_{S}=94.1\pm 1.5$ K and $T_{N}=85.7\pm 1.7$ K and no observable superconducting transition above 1.8 K.

**Fig. 1.**
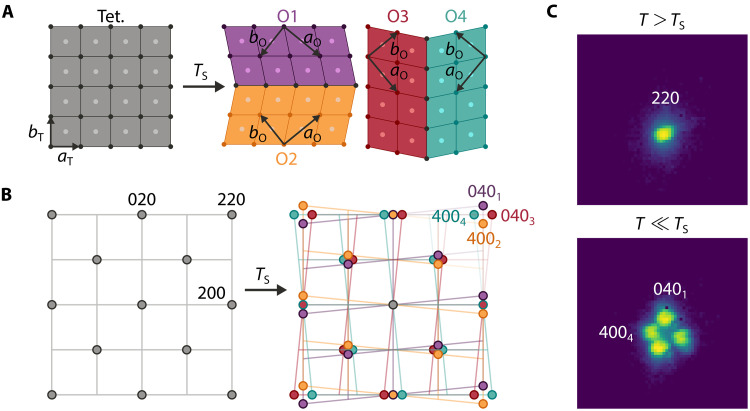
Signatures of the nematic structural phase transition in reciprocal space. (**A**) Left: Body-centered tetragonal crystal structure of Cu-Ba122 at high temperature viewed from the *c* axis. Right: Four possible face-centered orthorhombic domains formed as a result of the nematic structural phase transition at temperature $T_{S}≃94$ K. Orthorhombic primitive lattice vectors $a_{O}$ and $b_{O}$ are rotated by ≃45° with respect to tetragonal $a_{T}$ and $b_{T}$, where we denote $a_{O}$ as the long axis. (**B**) Reciprocal space picture of the structural phase transition. 220 reflection of the tetragonal phase splits into a quartet due to the formation of twin domains. Subscripts of the quartet reflections correspond to the different domains in (A). (**C**) Top: 220 reflection peak of Cu-Ba122 sample in tetragonal phase at T=100 K as measured by high-energy XRD. The incident beam is at the bottom left of the image relative to the peak. Bottom: Fully split 220 peak at base temperature T=10 K, where the peaks selected for DFXM measurements are labeled. The distortion of the quartet from the schematic in (B) is presumably due to the built-in strain and domain distribution as the sample is cooled through $T_{S}$ (*25*, *43*).

At room temperature, Cu-Ba122 has a tetragonal unit cell with space group *I*4/*mmm*. The ferroelastic phase transition at $T_{S}$ drives the crystal into an orthorhombic phase with space group *Fmmm*, in which the unit cell of the tetragonal phase develops a spontaneous symmetry-breaking strain along the ${\left[110\right]}_{T}$ direction–referred to as $\varepsilon_{\mathrm{xy}}$ or $\varepsilon_{B_{2g}}$ with respect to the tetragonal unit cell. This ferroelastic phase transition is a pseudoproper one: The critical order parameter is the electronic nematicity of $B_{2g}$ symmetry that also induces a spontaneous strain of the same symmetry through a bilinear nematoelastic coupling (*11*, *37*). The spontaneous strain can have a positive value in the ${\left[110\right]}_{T}$ or ${\left[1\bar{1}0\right]}_{T}$ direction, defining two orientational domain states. These two domain states can form twins along either of the two permissible domain wall directions of ${\left[100\right]}_{T}$ and ${\left[010\right]}_{T}$, leading to a total of four distinct suborientational orthorhombic domain types, as depicted in Fig. 1A. Furthermore, the unit cell in the orthorhombic phase is doubled and rotated by $45^{\circ }\pm \alpha$ with respect to that in the tetragonal phase, where $\alpha =\varepsilon_{\mathrm{xy}}\approx \frac{a_{O}-b_{O}}{a_{O}+b_{O}}$. We use the convention that $a_{O}$ ($b_{O}$) refers to the long (short) in-plane axis for each domain in the orthorhombic phase. The clapping angle α arises because the component domains of each twin rotate toward their corresponding twin boundary by α to stay in physical contact despite the orthorhombic distortion (*2*).

In real space, several surface- and bulk-sensitive methods have been applied to image the spatial dimensions of the orthorhombic domains in iron pnictides. Polarized light microscopy measurements have detected stripe-like domains elongated along the twin boundaries with domain widths of 5 to 100 μm in a variety of undoped, Co-doped, and P-doped BaFe_2_As_2_ samples (*26*, *38*–*40*). Moreover, bulk-sensitive measurements such as scanning superconducting quantum interference device microscopy (*41*) and quantum gas magnetometry (*40*) confirmed the dimensions observed in surface measurements and showed that the twin domains extend along the *c* axis into the bulk of the crystal. Despite the success of these techniques in detecting the shape and size of the twin domains in the orthorhombic phase, they all suffer from the shortcoming that their spatial resolution is at best several microns and cannot detect variations at that length scale. Moreover, none of the above techniques can directly probe bulk strain fields within the domains. While it is true that the angle of rotation of linearly polarized light upon reflection from a domain is proportional to the orthorhombic distortion (*40*), the skin depth of a few tens of nanometers in iron pnictides (*42*) renders optical microscopy measurements unable to probe the full depth of bulk samples with thicknesses of tens of microns.

In reciprocal space, the formation of ferroelastic twins below $T_{S}$ results in the splitting of the Bragg peaks of the tetragonal unit cell. Specifically, the $220_{T}$ reflection splits into four peaks with each peak corresponding to a different orthorhombic domain type, as demonstrated in Fig. 1B. Moreover, the splitting becomes larger at lower temperatures due to the growth of the degree of orthorhombicity and clapping angle as temperature is lowered. This peak splitting and the formation of twin domains were first observed in XRD studies on bulk samples of undoped and several Co-doping values of BaFe_2_As_2_ (*26*, *38*).

We confirmed this phenomenon in Cu-Ba122 in our preliminary reciprocal space measurement: Using high-energy XRD with a beam spot of 500 μm by 500 μm on a bulk single-crystal of Cu-Ba122, we observed a well-defined $220_{T}$ reflection of the tetragonal lattice at high temperatures. The $220_{T}$ reflection then splits at $T_{S}≃94$ K into four peaks as shown in Fig. 1C, indicating that the illuminated volume of the sample does contain all four types of domains in the orthorhombic phase. The slight distortion of the quartet peaks from a square can be explained by the built-in strain in the illuminated volume and the resulting domain distribution as the sample is cooled below $T_{S}$. Such distortions of the quartet pattern have been shown in undoped BaFe_2_As_2_ to be caused by domain realignment when external strain is applied on the sample in the ${\left[110\right]}_{T}$ direction (*25*, *43*). Because the illuminated volume in our measurement was freestanding, we ascribe the distortion in our measurement to built-in strain fields within the sample due to growth conditions.

### Measurement of strain waves using DFXM

**Fig. 2.**
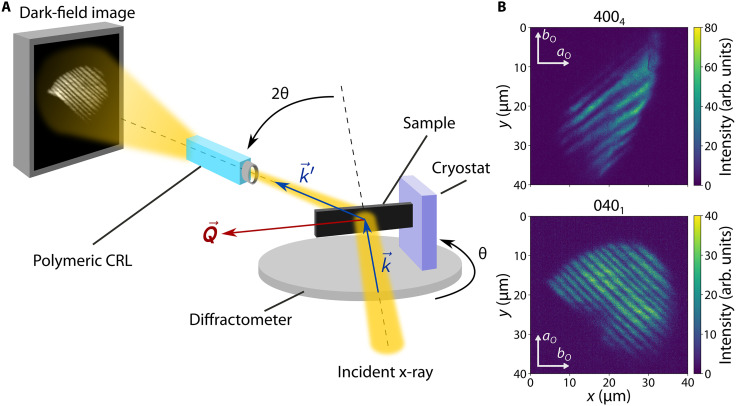
Imaging periodic scattering intensity modulations within individual nematic domains using DFXM. (**A**) DFXM experimental setup. Cu-Ba122 sample is mounted from one end on a cryostat installed on a horizontal diffractometer. A freestanding volume of the sample is illuminated by the incident x-ray beam in transmission geometry. A polymeric compound refractive lens (CRL) installed on the 2θ arm acts as an objective to image and magnify the scattered light emanating from the illuminated volume corresponding to a selected diffraction peak. The sample (θ) and the CRL (2θ) are rotated to capture the image of the desired Bragg peak (with scattering vector $\vec{Q}$) on the detector and also to scan the real-space distribution of axial strain and lattice orientation with respect to $\vec{Q}$. $\vec{k}$ and ${\vec{k}}^{\prime }$ correspond to the wave vectors of the incident and scattered radiation, respectively. (**B**) Real-space microscope images of the 400_4_ and 040_1_ peaks labeled in Fig. 1C. Each image depicts a single domain with mesoscopic spatial modulation of diffraction intensity, as explained in the main text. The above image was collected at T=3 K, and the one below at T=60 K. The curved outer perimeter of the images is due to a pinhole at the entrance of the objective lens. arb. units, arbitrary units.

DFXM is a full-field microscopy technique that enables the investigation of local spatial distribution of reciprocal space with the insertion of an x-ray objective lens in the path of the diffracted light (*27*, *44*–*46*). Our experimental setup for DFXM measurements consisted of a custom low-vibration cryostat installed on a diffractometer, a polymeric compound refractive lens (CRL) positioned on the 2θ-arm to image and magnify light diffracting at a particular scattering angle 2θ, and an area detector positioned on the image plane of the lens, as pictured in Fig. 2A (*30*, *47*, *48*). The small numerical aperture of the lens (*28*) in combination with a pinhole placed in front of the lens prevented stray diffraction and blurring. Additionally, a pinhole inserted in the back focal plane of the lens acted as a low-pass Fourier filter for off-axis diffraction. The insertion of the pinhole in the back focal plane further improved the high angular resolution of the lens thanks to its remarkably low numerical aperture.

The high angular resolution of the imaging system corresponds to a high resolution in reciprocal space and thus allows domain-selective imaging: Individual Bragg peaks in the orthorhombic phase can be selected to image the real-space distribution of the corresponding domain type. During our measurement, we imaged the 400_4_ and 040_1_ peaks as labeled in Fig. 1C; representative diffraction intensity images are given in Fig. 2B. The real-space images of scattering intensity show periodic stripe patterns along the ${\left[100\right]}_{T}$ and ${\left[010\right]}_{T}$ (or equivalently ${\left[110\right]}_{O}$ and ${\left[1\bar{1}0\right]}_{O}$) directions. The stripes in the $400_{4}$ image are along ${\left[100\right]}_{T}$ and those in the $040_{1}$ image are along ${\left[010\right]}_{T}$, which are the directions of the corresponding domain walls of the O4 and O1 domain types, respectively.

**Fig. 3.**
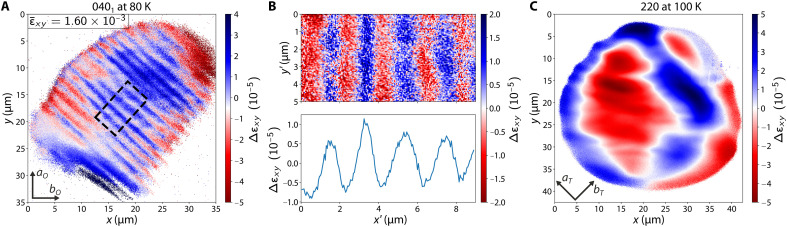
Detection of strain wave within a single nematic domain in the orthorhombic phase and loss of periodicity in the tetragonal phase. (**A**) Real-space strain map of a nematic domain scattering at the 040_1_ peak at 80 K. $\Delta \varepsilon_{\mathrm{xy}}$ refers to the relative strain with respect to the median $d_{040}$-spacing value of the depicted volume. ${\bar{\varepsilon }}_{\mathrm{xy}}$ is the average orthorhombicity measured at 80 K using conventional XRD. xy subscript corresponds to the diagonal direction of the *ab* face of the high-temperature tetragonal crystal structure. Dashed lines enclose a central area unaffected by vignetting effects at the boundaries, and the enclosed area is shown in more detail in (B). (**B**) Magnified view of the area enclosed by the dashed lines in (A). The primed axes $\left(x^{\prime },y^{\prime }\right)$ are rotated by 45° with respect to the unprimed axes (x,y). $\Delta \varepsilon_{\mathrm{xy}}$ is with respect to the median $d_{040}$-spacing value of the enclosed area. The plot below shows the values of $\Delta \varepsilon_{\mathrm{xy}}$ averaged along the $y^{\prime }$ direction in the plot above. We observe that the xy strain oscillates by ~10^−5^ with a wavelength of 2 to 3 μm. (**C**) Strain map of the 220 peak in the tetragonal phase at T=100 K. The spatial variation of strain lacks the coherence and periodicity observed in the nematic phase.

To identify the origin of the observed intensity stripes, we used (θ,2θ) scans, which yield real-space maps of strain along the axis of the scattering vector $\vec{Q}$, also referred to as axial strain. For our sample orientation, this strain corresponds to $\varepsilon_{\mathrm{xy}}$ with respect to the tetragonal unit cell. The high reciprocal-space resolution of the setup enabled not only the imaging of individual Bragg peaks but also of multiple distinct reciprocal-space points within a given Bragg peak. From Bragg’s law, reciprocal-space points with distinct 2θ values within a Bragg peak correspond to different interplanar spacing values (also known as *d*-spacing) between the parallel lattice planes associated with the imaged Bragg peak. As a result, regions with different *d*-spacings within a given domain were visualized. Resolving the fine *d*-spacing variations confirmed that the observed stripe-shaped intensity patterns arise from subdomain strain waves, as shown in Fig. 3A, rather than from switching between different domain types.

The observed strain waves have an amplitude of ~10^−5^ about the average *d*-spacing value of the given domain, a strain oscillation two orders of magnitude smaller than the ~10^−3^ strain difference between the component domains of a twin, verifying that the observation is a monodomain phenomenon. Moreover, the oscillations have a wavelength at the mesoscopic scale of ~2 μm and permeate the imaged section of the entire domain, as shown in the zoomed image of Fig. 3B.

Upon entering the tetragonal phase, a marked change happens in the strain map of the sample. As the orthorhombicity disappears, so do the separate domains, and the quartet of peaks merges back into a single $220_{T}$ peak. Performing a (θ,2θ) scan on this peak reveals a strain distribution that lacks the periodic wave pattern observed in the low-temperature orthorhombic phase, as depicted in Fig. 3C. We infer from these results that the strain waves observed below $T_{S}$ are purely a phenomenon of the low-temperature nematic phase, where the emergence of orthorhombic twin domain walls along symmetry-allowed directions also sets the directionality of the observed strain wave.

### Orientation maps and determination of the wavelength of strain waves

**Fig. 4.**
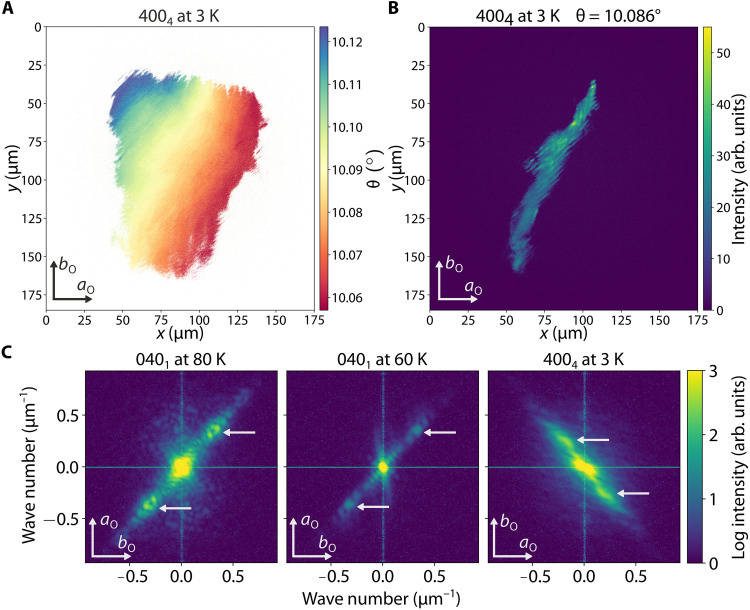
Lack of lattice tilt contribution to intensity modulations and negligible dependence of wavelength on temperature. (**A**) Large area spatial map of local orientation of the (400)_4_ lattice planes as determined by the center of mass of the intensity distribution as a function of θ at each individual detector pixel. θ varies by ~0.06° over the large length scale of ~100 μm and there are no observable orientation modulations with wavelength of 2 to 3 μm. (**B**) Large area map of scattering intensity at a particular lattice orientation value θ=10.086°. Intensity modulations with wavelength of 2 to 3 μm are visible across a length scale of 100 μm. This intensity map is part of the dataset used to determine the local orientation map of (A) and demonstrates that intensity modulations are observable in a region where orientation does not change appreciably. (**C**) Fourier transforms (FTs) of microscope images of the 040_1_ and 400_4_ peaks at various temperatures. The white arrows highlight the main satellite peaks corresponding to the wavenumber of the intensity modulations. arb. units, arbitrary units.

To further understand the phenomenon of strain waves, we also investigate how lattice orientation is affected by the presence of $\varepsilon_{\mathrm{xy}}$ strain waves. DFXM not only enables us to measure the local variation of axial strain but also permits the determination of the spatial dependence of the lattice tilt or orientation. The lattice orientation by itself does not directly measure a particular strain value; however, its continuous variation corresponds to a combination of out-of-plane strain components. We measured the lattice orientation by performing a fine θ scan at a fixed scattering angle 2θ in the vicinity of a selected Bragg peak. Moreover, we performed this measurement at various adjacent sites on the sample to cover a region of ~100 μm by 100 μm. The resulting orientation map reveals a gradual and approximately monotonic evolution of the lattice tilt across a large area without a marked stripe pattern, as depicted in Fig. 4A. The longer length-scale variation in orientation is likely due to a slight bend of the lattice in the imaged volume within the crystal and appears unrelated to the detected strain waves. In contrast, the intensity image corresponding to a specific lattice tilt value used in the orientation map (Fig. 4B) exhibits pronounced stripes, indicating that the observed intensity stripes occur independently of any local variations in lattice orientation. This observation further suggests that the diffraction intensity stripes are caused predominantly by $\varepsilon_{\mathrm{xy}}$ strain waves.

With the existence of $\varepsilon_{\mathrm{xy}}$ strain waves established, we next determine that the wavelength of these waves do not change appreciably as a function of temperature. As we have already shown that the intensity stripes are caused by strain waves, we used the wavelength of the intensity stripes as a substitute for the wavelength of the strain waves. We performed a Fourier transform (FT) on individual images taken at a given temperature and summed the absolute value of these FTs to determine an aggregate wavelength value for that temperature. The resulting two-dimensional FT figures plotting the salient wave vectors at three different temperatures are shown in Fig. 4C. The figures are plotted in a logarithmic intensity scale to distinguish the finite wave vector peaks of interest that are orders of magnitude smaller in intensity compared to the zero-wave vector peak. This large central peak is due to the positive average value of intensity in each image. The main sinusoidal satellite peaks with the highest intensity are indicated by arrows in Fig. 4C and correspond to the wavelength of ~2 μm observed in the real-space images shown in previous figures. As expected, the satellite peaks of the 040_1_ and 400_4_ Bragg peaks lie on axes rotated by 90° with respect to each other. A numerical investigation of the wavelength corresponding to these main satellite peaks reveals that the wavelength does not change in any appreciable way as a function of temperature. Besides the main satellite peaks, we also observe a set of quasi-periodic subdominant peaks in the FT images, which we discuss in the Supplementary Text.

### Compatibility relations set the wave direction

The phenomenon of strain waves within bulk nematic domains of an iron pnictide that we have presented has not yet been observed in any other class of quantum materials to our knowledge and challenges the conventional understanding of how strain behaves mesoscopically in materials with structural distortions. This result demonstrates that spatially coherent strain fields can emerge inside structural domains, and it is essential to model strain as a local field within materials rather than a uniform value. This insight is important, as the intuition of homogeneous strains developed at the macroscopic level does not necessarily apply to strain fields varying at the mesoscopic level.

Unlike homogeneous strains, locally varying strain fields must obey certain constraints. From a geometrical perspective, continuous deformations of a material will displace individual unit cells from their equilibrium positions, r, to a new position, $r^{\prime }=r+u\left(r\right)$. Thus, the three-component displacement vector, u(r), represents the complete description of the deformation. The six-component strain tensor, $\varepsilon_{\mathrm{ij}}\left(r\right)$, is overcomplete, because its six components are various derivatives of the three components of u(r). The constraints on the strain tensor reduce the number of independent strain components from six in the homogeneous limit to only three inhomogeneous ones. For infinitesimal strains, $2\varepsilon_{\mathrm{ij}}\left(r\right)={\text{∂}}_{j}u_{i}\left(r\right)+{\text{∂}}_{i}u_{j}\left(r\right)$, which results in constraints known as the Saint-Venant compatibility relations (SVCRs) of continuum elasticity theory (*49*–*51*). The impact of the SVCR on martensite and ferroelectric domains has been previously discussed (*52*, *53*); here, we focus on its effect on nematic domains.

**Fig. 5.**
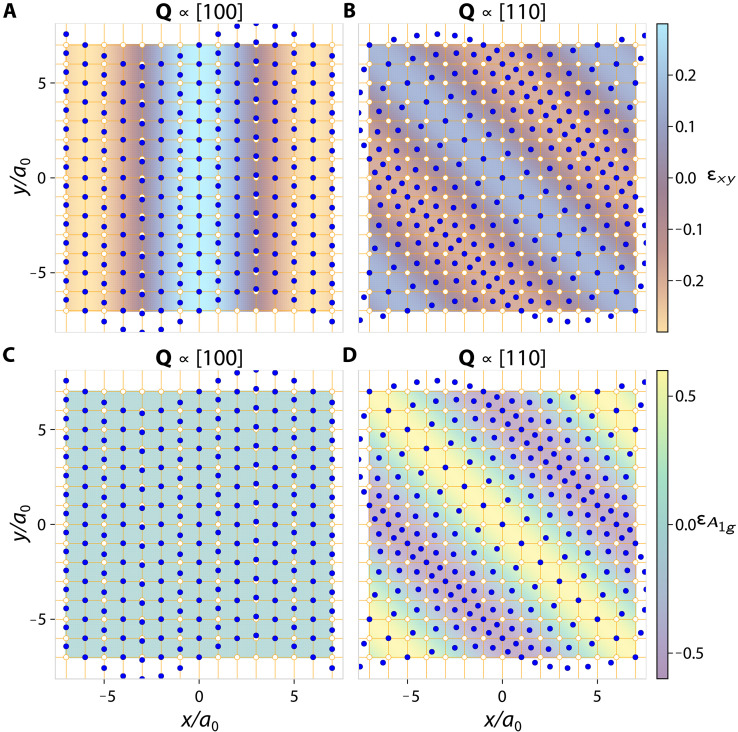
Static shear waves constrained by SVCRs. A square lattice, with equilibrium positions (orange, open circles) and lattice constant $a_{0}$, which is deformed by a static shear wave $\left(\varepsilon_{\mathrm{xy}}\right)$ into displaced positions (blue closed circles). By satisfying the Saint-Venant compatibility relation (SVCR) in Eq. 1, the shear waves with arbitrary momenta will result in lattice displacements that may simultaneously induce volume-changing, energetically costly, dilatation strain: $\varepsilon_{A_{1g}}\equiv \varepsilon_{\mathrm{xx}}+\varepsilon_{\mathrm{yy}}$. (**A** and **B**) Shear waves with wave vector Q∝[100] and Q∝[110], respectively, and (**C** and **D**) the corresponding dilatation waves generated by them. The pair (A and C) [also the pair (B and D)] has exactly the same displacement vector field, u(r), integrated from a compatible shear wave, $\varepsilon_{\mathrm{xy}}\left(r\right)\propto \text{cos}\left(Q\cdot r\right)$. When Q∝[100] (A and C), the dilatation strain vanishes, whereas when Q∝[110] (B and D), the dilatation is maximal. The strain waves in this figure are depicted with a zero uniform shear strain background, whereas the nematic domains in the material studied have uniform shear strain values (~10^−3^) much larger than the amplitude of the measured modulation (~10^−5^). As discussed in the main text, only the nonuniform part of the shear strain induces a dilatation. The distortions depicted in this figure are exaggerated with respect to the lattice constant for clarity ($\varepsilon_{\mathrm{xy}}$ ~ 0.2 as opposed to the measured $\varepsilon_{\mathrm{xy}}$ ~ 10^−5^ in Fig. 3).

Consequently, for nonuniform strains, distinct irreducible representations of the strain tensor are coupled to each other despite being orthogonal in symmetry. In systems with $D_{4h}$ point group symmetry undergoing planar deformations, the three nonzero strain components—the dilatation $\varepsilon_{A_{1g}}\equiv \varepsilon_{\mathrm{xx}}+\varepsilon_{\mathrm{yy}}$, the deviatoric $\varepsilon_{B_{1g}}\equiv \varepsilon_{\mathrm{xx}}-\varepsilon_{\mathrm{yy}}$, and the shear $\varepsilon_{B_{2g}}\equiv 2\varepsilon_{\mathrm{xy}}$ strain—are all related by a single SVCR

$$\left(\partial_{x}^{2}+\partial_{y}^{2}\right)\varepsilon_{A_{1g}}\left(r\right)=\left(\partial_{x}^{2}-\partial_{y}^{2}\right)\varepsilon_{B_{1g}}\left(r\right)+\left(2\partial_{x}\partial_{y}\right)\varepsilon_{B_{2g}}\left(r\right)$$ (1)

which is straightforwardly proven from the definition of the infinitesimal strain tensor. Whereas the dilatation is a symmetry-preserving, volume-changing strain, both the deviatoric and shear are symmetry-breaking, but volume-preserving, strains. The SVCR above shows that mesoscopic shear ($\varepsilon_{B_{2g}}$) modulations incur an additional elastic energy associated with dilatation ($\varepsilon_{A_{1g}}$) and thus related to the bulk modulus, unless they are waves with momentum lying along the ${\left[100\right]}_{T}$ or ${\left[010\right]}_{T}$ axes. We demonstrate this constraint in Fig. 5, where we show that the interdependence between the shear and dilatation waves is controlled by the momentum direction. In Fig. 5 (A and C), a compatible shear wave with momentum along ${\left[100\right]}_{T}$ induces a displacement vector that has no dilatation strain, whereas, when the momentum is along ${\left[110\right]}_{T}$, the associated displacement vector triggers local volume changes that maximize the dilatation strain (Supplementary Text).

Whereas the strain basis is adopted from the crystalline point group $D_{4h}$, Eq. 1 is a fundamental constraint imposed by geometry and therefore applies for media of any symmetry or elastic stiffness (*50*, *51*). Thus, there is a particular universality for strain waves in materials near tetragonal-to-orthorhombic ferroelastic transitions. Regardless of the specific elastic properties of a tetragonal crystal, $\varepsilon_{\mathrm{xy}}$ spatial fluctuations above the ferroelastic transition will generally induce energetically expensive dilatations unless the wave vector lies along the in-plane crystal axes. The softest fluctuations above the transition will then also be those without dilatations, and the modes with momenta pointing along the in-plane crystal axes will be the first to freeze into static modulations below the transition (*50*, *51*, *54*).

### Proposed mechanism for the strain waves in Cu-Ba122

The SVCR in Eq. 1 provides a general geometric reason for why we would expect $B_{2g}$ strain waves to modulate along the ${\left[100\right]}_{T}$/${\left[010\right]}_{T}$ axes if such modulations do exist in the crystal. Here, we further propose a minimal real-space elastostatic model that attempts to address why it would be energetically favorable for periodic modulations to arise in the examined twin domains in the first place. Without any pretense that this model is the exact explanation for our observations, we show that, if the twin is modeled as a one-dimensional object, restricted in direction by the SVCR, then uniform twin domains with a sharp twin boundary are unstable toward spatial modulations. Moreover, the characteristic modulation wavelength Λ emerges as a length scale almost an order of magnitude larger than the mean-field correlation length of the order parameter, $\xi_{\text{MF}}$, yet is still orders of magnitude smaller than the size of the twin, *L*, away from the critical region.

First and foremost, the restriction of the modulation momentum to the in-plane crystal axes renders the two-dimensional nemato-elastic problem effectively one-dimensional. For concreteness, we consider modulations along the ${\left[100\right]}_{T}$ direction. For modulations along this direction, there is a local proportionality between the shear strain, $\varepsilon_{\mathrm{xy}}\left(r\right)$, and the $B_{2g}$ electronic nematic order parameter, ϕ(r). Consequently, the complexity associated with solving coupled nonlocal equations for spatially modulated strain and nematic fields is reduced to only solving for a single field in the one-dimensional limit (Supplementary Text).

In the absence of external strain fields, the traction-free boundary conditions from elasticity theory (Supplementary Text) coupled with nemato-elasticity require that ϕ(x)→0 on the outer boundaries of the twins, modeled here at x=0 and x=L. The twinning, however, adds the constraints that the electronic nematic order parameter is finite and of opposite signs within the bulk of each twin component and goes through zero at the twin boundary. Thus, we are left to solve a constrained Ginzburg-Landau minimization problem along the *x* axis, modeling the nematic free energy as the usual $\phi^{4}$ model with the following one-dimensional free energy functional

$$\begin{array}{c} F\left[\phi \left(x\right)\right]\\ =\frac{1}{2}\int_{0}^{L}\mathrm{dx}\;\left\{a\left(T-T^{*}\right)\phi^{2}\left(x\right)+{\left[{\text{∂}}_{x}\phi \left(x\right)\right]}^{2}\right\}+u\int_{0}^{L}\mathrm{dx}\;\phi^{4}\left(x\right) \end{array}$$ (2)

for field configurations, ϕ(x), satisfying the stated constraints. In the above expression, *L* is the size of the two twin domains, a>0 is the coefficient of the inverse susceptibility as a function of temperature (T), $T^{*}$ is the nematic critical temperature, and u>0 provides thermodynamic stability. $T^{*}$ in our model corresponds to the experimentally determined structural transition temperature $T_{S}$ in the material. The free energy is given in units where the nematic stiffness is 1. Given that the stated boundary conditions overdetermine the problem, we use a variational approach to obtain a metastable ansatz that admits modulations within the twin domains.

From a macroscopic perspective, we expect twin domains to be uniform within the bulk, separated by sharp domain walls. Intuitively, a square wave solution satisfies the prescribed boundary conditions and should be thermodynamically stable. We therefore choose the physically plausible ansatz of a partial Fourier series of the square wave, which both has the expected limiting behavior and allows for spatial modulations

$$\phi_{M}\left(s\right)\equiv \Phi \cdot \frac{4}{\pi }\sum_{m\;\text{odd}}^{M}\frac{\text{sin}\left(2\pi \mathrm{ms}\right)}{m},\;s\equiv \frac{x}{L}\in \left(0,1\right)$$ (3)

where the amplitude, Φ, and the upper bound of the summation, *M*, are variational parameters. The first limit, M=1, corresponds to a single sinusoid of wavelength, *L*. The second limit, M→∞, meanwhile, corresponds to flat twin domains with an infinitely sharp domain wall between the two. Whereas neither limit has spatial modulations within either twin domain, any M∈(1,∞) solution will contain bulk modulations. As shown in the Supplementary Materials, the value of *M* that minimizes the free-energy functional is finite below the transition temperature, indicating that spatially modulated twin domains are energetically favored over uniform twin domains separated by a sharp boundary. Moreover, we show that our model predicts both the wavelength and amplitude of the spatial modulations to slowly decrease within the ordered phase, scaling as $1/\sqrt{T^{*}-T}$. Because our data were collected at temperatures not in the immediate vicinity of $T_{S}≃94$ K, the model is consistent with our observation that the modulation wavelength does not change appreciably as a function of the probed temperatures.

## DISCUSSION

Our results demonstrate the power of DFXM as a tool for probing mesoscopic strain fields in quantum materials, where structural distortions can have important effects on functional, electronic, and magnetic properties. Mesoscopic phenomena are prevalent in strongly correlated systems (*55*), with strain and phase separation affecting transport (*56*) and superconducting behavior (*57*) of a variety of complex oxides. With the installation of fourth-generation synchrotrons, advances in x-ray optics, and the development of novel depth-sensitive imaging techniques such as coded-aperture imaging (*58*), DFXM will be well positioned to shed light on the structural properties of many complex materials.

Our measurement also opens fresh real-space perspectives on the relation between the electronic and lattice degrees of freedom in iron-based superconductors. These materials exhibit strong nemato-elastic coupling (*59*), and the pronounced elastoresistive response reported in prior measurements (*17*, *37*) suggests that the strain wave amplitude of 10^−5^ observed in our results could induce substantial spatial variations in the local resistivity anisotropy within individual domains. However, it remains unknown whether, or how, this phenomenon influences superconductivity in these materials. As the orthorhombic order parameter is suppressed by increased doping, our model predicts a corresponding suppression of the strain wave amplitude within domains. Intriguingly, in Ba(Fe_1−*x*_Co*_x_*)_2_As_2_, it has been shown that, as doping approaches the optimal value for superconductivity from the underdoped regime, the nemato-elastic coupling weakens while the nematic scattering cross section increases markedly (*60*). Given that superconducting pairing is strongly enhanced by nematic fluctuations near a nematic quantum critical point (*61*) and that strain can suppress superconductivity by damping these fluctuations (*12*), investigating how such strain waves evolve with doping is essential for understanding their impact on superconductivity.

## MATERIALS AND METHODS

### Growth and characterization of single crystals

Single crystals of Ba(Fe_1−*x*_Cu*_x_*)_2_As_2_ were grown using an FeAs flux as described elsewhere (*32*, *35*). The crystals grow with a plate-like morphology, where the large faces correspond to the *ab* plane of the tetragonal lattice. The sample used for the DFXM measurements in this work was taken from a growth batch with a Cu composition of x=0.02±0.0008 as determined by electron probe microanalysis wavelength-dispersive spectroscopy using parent compound BaFe_2_As_2_ and elemental Cu metal as calibration samples. The measured sample was cleaved from a single crystal using a razor blade, and it had the dimensions 3.9 mm by 1.4 mm by 85 μm.

We established the mean and SD of the Cu concentration of the batch by performing WDS on 11 freshly cleaved samples chosen from various single crystals within the batch in a JEOL JXA-8230 “SuperProbe” electron microprobe. Ten distinct ~1-μm-sized spots across each of the 11 samples were selected for measurements to estimate the spatial and statistical variation of Cu concentration within individual samples and across the batch. No systematic spatial variation of Cu concentration was detected, and the SD was found to be 0.0008 across 11 distinct samples with a mean Cu concentration of x=0.02.

Four-point resistivity measurements were performed on five samples from the same batch to estimate the mean transition temperatures $T_{S}$ and $T_{N}$ for the structural transition and the Néel transition of the batch, respectively. It has been experimentally shown that the Fisher-Langer relation $C_{p}^{\left(c\right)}\propto \frac{\partial \rho^{\left(c\right)}}{\partial T}$ between the critical anomaly in the heat capacity and that in the temperature derivative of resistivity holds in the vicinity of both second-order phase transitions in this material class (*62*). From the resistivity derivative measurements, we determined that for this batch $T_{S}=94.1\pm 1.5$ K and $T_{N}=85.7\pm 1.7$ K. The sample investigated here was also confirmed to have $T_{S}=94\pm 1.5$ K using XRD as elaborated below.

### High-energy XRD

Before measuring the sample in DFXM, we performed high-energy XRD on the sample at Beamline 6-ID-D of the Advanced Photon Source, Argonne National Laboratory, USA. The sample was glued from its corner on a copper mount with its [100] crystal axis aligned vertically and positioned on a Huber six-circle diffractometer such that a large freestanding sample volume was at the center of rotation. We illuminated the sample with a monochromatic x-ray beam with beam size 500 μm by 500 μm and x-ray energy of 87 keV. A Dectris Pilatus 2M CdTe area detector was positioned 2.6 m away from the sample and off the beam axis to capture the tetragonal 220 peak with high resolution. The sample was kept under vacuum inside a Kapton dome and cooled from 300 K down to 10 K using a cryocooler in steps of 2 K. At each temperature step, the sample was rotated around the vertical axis, and the diffracted light was collected. Raw detector images were used to establish that the 220 peak splitting occurred at $T_{S}≃94$ K.

### Dark-field x-ray microscopy

We performed the DFXM measurements at Beamline 6-ID-C of the Advanced Photon Source. The sample was mounted on the sample holder of a customized low-vibration Montana Instruments s100 helium cryostat, which itself was mounted on a diffractometer via a vibration-damped optical breadboard (*30*). The sample was kept under vacuum in a chamber with beryllium entry and exit windows aligned for a horizontal scattering geometry. The sample was glued onto the sample holder by GE-7031 varnish from one of its corners such that an area of ~3.2 mm by 1 mm was freestanding and available for measurement in the transmission geometry. We performed measurements on a volume of this freestanding part of the sample ~2.5 to 2.7 mm away from the glued part, therefore, far away from any region that might be affected by the differential thermal contraction from the sample holder. The sample was aligned such that the [110] direction of the crystal axes lay within the horizontal scattering plane.

We used a monochromatic beam of x-rays tuned to 20 keV by a Si (111) double-crystal monochromator to illuminate our sample. At this energy, the x-ray attenuation length estimated for BaFe_2_As_2_ is ~80 μm, which is approximately the thickness of our sample. The x-ray beam was focused on the sample using a Beryllium CRL, resulting in an illuminated area of ~35 μm by 35 μm on the sample with high photon flux density. Downstream of the sample a 50-μm pinhole and a polymeric CRL were positioned on the 2θ arm of the diffractometer to image the light diffracted at a particular scattering angle while blocking out other divergent scattered light. The polymeric CRL was designed for use at 20 keV and has a focal length of 131 mm at that x-ray energy (*28*); it acted as our objective lens and had a working distance of 140 mm from the sample. Another 50-μm pinhole was positioned at the back focal plane of the objective lens to filter out light originating from off-angle scattering. The small numerical aperture of the objective lens in combination with the pinholes led to an angular resolution of ~0.001°.

We used a high-resolution area detector placed ≃2.435 m downstream from the sample at the image plane to collect the x-ray microscopy images. The detector is composed of a 10-μm-thick LuAG:Ce scintillator that converts x-rays to visible light, a 5× optical objective lens, and an Andor 5.5-megapixel sCMOS Zyla camera. At this configuration, the x-ray magnification from the polymeric CRL objective lens was ×26, which, combined with the detector’s ×5 optical magnification, led to a total magnification of ×130. Given the 6.5 μm–by–6.5 μm pixel size of the Zyla detector, this total magnification yielded an effective pixel size of 50 nm in our microscopy images. The microscope’s resolution was determined to be 300 nm. The field of view was determined by the illuminated area and had dimensions of ~35 μm by 35 μm.

## Supplementary Materials

### This PDF file includes:

Supplementary Text Figs. S1 to S7 References

## References

[1] E. K. H. Salje, Ferroelastic materials. Annu. Rev. Mat. Res. 42, 265–283 (2012).

[2] A. K. Tagantsev, L. E. Cross, J. Fousek, *Domains in Ferroic Crystals and Thin Films* (Springer, ed. 1, 2010).

[3] E. K. H. Salje, *Phase Transitions in Ferroelastic and Co-elastic Crystals* (Cambridge Univ. Press, 1993).

[4] S. A. Kivelson, E. Fradkin, V. J. Emery, Electronic liquid-crystal phases of a doped Mott insulator. Nature 393, 550–553 (1998).

[5] E. Fradkin, S. A. Kivelson, M. J. Lawler, J. P. Eisenstein, A. P. Mackenzie, Nematic Fermi fluids in condensed matter physics. Annu. Rev. Condens. Matter Phys. 1, 153–178 (2010).

[6] R. M. Fernandes, A. V. Chubukov, J. Schmalian, What drives nematic order in iron-based superconductors? Nat. Phys. 10, 97–104 (2014).

[7] M. J. Hÿtch, J.-L. Putaux, J.-M. Pénisson, Measurement of the displacement field of dislocations to 0.03 Å by electron microscopy. Nature 423, 270–273 (2003). 12748637 10.1038/nature01638

[8] M. J. Hÿtch, E. Snoeck, R. Kilaas, Quantitative measurement of displacement and strain fields from HREM micrographs. Ultramicroscopy 74, 131–146 (1998).

[9] H. Simons, A. B. Haugen, A. C. Jakobsen, S. Schmidt, F. Stöhr, M. Majkut, C. Detlefs, J. E. Daniels, D. Damjanovic, H. F. Poulsen, Long-range symmetry breaking in embedded ferroelectrics. Nat. Mater. 17, 814–819 (2018). 29941920 10.1038/s41563-018-0116-3

[10] J. Chu, J. G. Analytis, K. De Greve, P. L. McMahon, Z. Islam, Y. Yamamoto, I. R. Fisher, In-Plane resistivity anisotropy in an underdoped iron arsenide superconductor. Science 329, 824–826 (2010). 20705856 10.1126/science.1190482

[11] J. H. Chu, H. H. Kuo, J. G. Analytis, I. R. Fisher, Divergent nematic susceptibility in an iron arsenide superconductor. Science 337, 710–712 (2012). 22879513 10.1126/science.1221713

[12] P. Malinowski, Q. Jiang, J. J. Sanchez, J. Mutch, Z. Liu, P. Went, J. Liu, P. J. Ryan, J.-W. Kim, J.-H. Chu, Suppression of superconductivity by anisotropic strain near a nematic quantum critical point. Nat. Phys. 16, 1189–1193 (2020).

[13] A. Steppke, L. Zhao, M. E. Barber, T. Scaffidi, F. Jerzembeck, H. Rosner, A. S. Gibbs, Y. Maeno, S. H. Simon, A. P. Mackenzie, C. W. Hicks, Strong peak in *T*_c_ of Sr_2_RuO_4_ under uniaxial pressure. Science 355, eaaf9398 (2017). 28082534 10.1126/science.aaf9398

[14] J. Straquadine, M. Ikeda, I. Fisher, Evidence for realignment of the charge density wave state in ErTe_3_ and TmTe_3_ under uniaxial stress via elastocaloric and elastoresistivity measurements. Phys. Rev. X 12, 021046 (2022).

[15] V. Sunko, E. Abarca Morales, I. Marković, M. E. Barber, D. Milosavljević, F. Mazzola, D. A. Sokolov, N. Kikugawa, C. Cacho, P. Dudin, H. Rosner, C. W. Hicks, P. D. C. King, A. P. Mackenzie, Direct observation of a uniaxial stress-driven Lifshitz transition in Sr_2_RuO_4_. NPJ Quantum Mater. 4, 46 (2019).

[16] J. Mutch, W.-C. Chen, P. Went, T. Qian, I. Z. Wilson, A. Andreev, C.-C. Chen, J.-H. Chu, Evidence for a strain-tuned topological phase transition in ZrTe_5_. Sci. Adv. 5, eaav9771 (2019). 31448327 10.1126/sciadv.aav9771 PMC6688871

[17] H. H. Kuo, J. H. Chu, J. C. Palmstrom, S. A. Kivelson, I. R. Fisher, Ubiquitous signatures of nematic quantum criticality in optimally doped Fe-based superconductors. Science 352, 958–962 (2016). 27199422 10.1126/science.aab0103

[18] C. W. Hicks, M. E. Barber, S. D. Edkins, D. O. Brodsky, A. P. Mackenzie, Piezoelectric-based apparatus for strain tuning. Rev. Sci. Instrum. 85, 065003 (2014). 24985843 10.1063/1.4881611

[19] J. J. Sanchez, P. Malinowski, J. Mutch, J. Liu, J.-W. Kim, P. J. Ryan, J.-H. Chu, The transport-structural correspondence across the nematic phase transition probed by elasto X-ray diffraction. Nat. Mater. 20, 1519–1524 (2021). 34446865 10.1038/s41563-021-01082-4

[20] A. G. Singh, M. D. Bachmann, J. J. Sanchez, A. Pandey, A. Kapitulnik, J. W. Kim, P. J. Ryan, S. A. Kivelson, I. R. Fisher, Emergent tetragonality in a fundamentally orthorhombic material. Sci. Adv. 10, eadk3321 (2024). 38781340 10.1126/sciadv.adk3321 PMC11114214

[21] M. D. Bachmann, G. M. Ferguson, F. Theuss, T. Meng, C. Putzke, T. Helm, K. R. Shirer, Y. S. Li, K. A. Modic, M. Nicklas, M. König, D. Low, S. Ghosh, A. P. Mackenzie, F. Arnold, E. Hassinger, R. D. McDonald, L. E. Winter, E. D. Bauer, F. Ronning, B. J. Ramshaw, K. C. Nowack, P. J. Moll, Spatial control of heavy-fermion superconductivity in CeIrIn_5_. Science 366, 221–226 (2019). 31601766 10.1126/science.aao6640

[22] P. J. Moll, Focused ion beam microstructuring of quantum matter. Annu. Rev. Condens. Matter Phys. 9, 147–162 (2018).

[23] D. A. Freedman, D. Roundy, T. A. Arias, Elastic effects of vacancies in strontium titanate: Short- and long-range strain fields, elastic dipole tensors, and chemical strain. Phys. Rev. B 80, 064108 (2009).

[24] W. W. Schmahl, A. Putnis, E. Saue, P. Freeman, A. Graeme-Barber, R. Jones, K. K. Singh, J. Blunt, P. P. Edwards, J. Loram, K. Mirza, Twin formation and structural modulations in orthorhombic and tetragonal YBa_2_(Cu_1-x_Co_x_)_3_O_7-δ_. Philos. Mag. Lett. 60, 241–248 (1989).

[25] E. C. Blomberg, A. Kreyssig, M. A. Tanatar, R. M. Fernandes, M. G. Kim, A. Thaler, J. Schmalian, S. L. Bud’ko, P. C. Canfield, A. I. Goldman, R. Prozorov, Effect of tensile stress on the in-plane resistivity anisotropy in BaFe_2_As_2_. Phys. Rev. B 85, 144509 (2012).

[26] R. Prozorov, M. A. Tanatar, N. Ni, A. Kreyssig, S. Nandi, S. L. Bud’ko, A. I. Goldman, P. C. Canfield, Intrinsic pinning on structural domains in underdoped single crystals of Ba(Fe_1−*x*_Co*_x_*)_2_As_2_. Phys. Rev. B 80, 174517 (2009).

[27] H. Simons, A. King, W. Ludwig, C. Detlefs, W. Pantleon, S. Schmidt, F. Stöhr, I. Snigireva, A. Snigirev, H. F. Poulsen, Dark-field X-ray microscopy for multiscale structural characterization. Nat. Commun. 6, 6098 (2015). 25586429 10.1038/ncomms7098 PMC4354092

[28] Z. Qiao, X. Shi, P. Kenesei, A. Last, L. Assoufid, Z. Islam, A large field-of-view high-resolution hard x-ray microscope using polymer optics. Rev. Sci. Instrum. 91, 113703 (2020). 33261446 10.1063/5.0011961

[29] L. E. Dresselhaus-Marais, G. Winther, M. Howard, A. Gonzalez, S. R. Breckling, C. Yildirim, P. K. Cook, M. Kutsal, H. Simons, C. Detlefs, J. H. Eggert, H. F. Poulsen, In situ visualization of long-range defect interactions at the edge of melting. Sci. Adv. 7, eabe8311 (2021). 34261647 10.1126/sciadv.abe8311 PMC8279502

[30] J. Plumb, I. Poudyal, R. L. Dally, S. Daly, S. D. Wilson, Z. Islam, Dark field X-ray microscopy below liquid-helium temperature: The case of NaMnO_2_. Mater Charact 204, 113174 (2023).

[31] J. Plumb, A. C. Salinas, K. Mallayya, E. Kisiel, F. B. Carneiro, R. Gomez, G. Pokharel, E.-A. Kim, S. Sarker, Z. Islam, S. Daly, S. D. Wilson, Phase-separated charge order and twinning across length scales in CsV_3_Sb_5_. Phys. Rev. Mater. 8, 093601 (2024).

[32] J.-H. Chu, J. G. Analytis, C. Kucharczyk, I. R. Fisher, Determination of the phase diagram of the electron-doped superconductor Ba(Fe_1−*x*_Co*_x_*)_2_As_2_. Phys. Rev. B 79, 014506 (2009).

[33] J. Paglione, R. L. Greene, High-temperature superconductivity in iron-based materials. Nat. Phys. 6, 645–658 (2010).

[34] R. M. Fernandes, A. I. Coldea, H. Ding, I. R. Fisher, P. J. Hirschfeld, G. Kotliar, Iron pnictides and chalcogenides: A new paradigm for superconductivity. Nature 601, 35–44 (2022). 34987212 10.1038/s41586-021-04073-2

[35] N. Ni, A. Thaler, J. Q. Yan, A. Kracher, E. Colombier, S. L. Bud’ko, P. C. Canfield, S. T. Hannahs, Temperature versus doping phase diagrams for Ba(Fe_1−*x*_TM*_x_*)_2_As_2_ (TM = Ni, Cu, Cu/Co) single crystals. Phys. Rev. B 82, 024519 (2010).

[36] R. M. Fernandes, M. G. Vavilov, A. V. Chubukov, Enhancement of *T_c_* by disorder in underdoped iron pnictide superconductors. Phys. Rev. B 85, 140512 (2012).

[37] H. H. Kuo, M. C. Shapiro, S. C. Riggs, I. R. Fisher, Measurement of the elastoresistivity coefficients of the underdoped iron arsenide Ba(Fe_0.975_Co_0.025_)_2_As_2_. Phys. Rev. B 88, 085113 (2013).

[38] M. A. Tanatar, A. Kreyssig, S. Nandi, N. Ni, S. L. Bud’ko, P. C. Canfield, A. I. Goldman, R. Prozorov, Direct imaging of the structural domains in the iron pnictides *A*Fe_2_As_2_ (*A* = Ca, Sr, Ba). Phys. Rev. B 79, 180508 (2009).

[39] E. Thewalt, I. M. Hayes, J. P. Hinton, A. Little, S. Patankar, L. Wu, T. Helm, C. V. Stan, N. Tamura, J. G. Analytis, J. Orenstein, Imaging anomalous nematic order and strain in optimally doped BaFe_2_(As,P)_2_. Phys. Rev. Lett. 121, 027001 (2018). 30085755 10.1103/PhysRevLett.121.027001

[40] F. Yang, S. F. Taylor, S. D. Edkins, J. C. Palmstrom, I. R. Fisher, B. L. Lev, Nematic transitions in iron pnictide superconductors imaged with a quantum gas. Nat. Phys. 16, 514–519 (2020).

[41] B. Kalisky, J. R. Kirtley, J. G. Analytis, J.-H. Chu, A. Vailionis, I. R. Fisher, K. A. Moler, Stripes of increased diamagnetic susceptibility in underdoped superconducting Ba(Fe_1−*x*_Co*_x_*)_2_As_2_ single crystals: Evidence for an enhanced superfluid density at twin boundaries. Phys. Rev. B 81, 184513 (2010).

[42] L. Stojchevska, T. Mertelj, J.-H. Chu, I. R. Fisher, D. Mihailovic, Doping dependence of femtosecond quasiparticle relaxation dynamics in Ba(Fe,Co)_2_As_2_ single crystals: Evidence for normal-state nematic fluctuations. Phys. Rev. B 86, 024519 (2012).

[43] M. A. Tanatar, E. C. Blomberg, A. Kreyssig, M. G. Kim, N. Ni, A. Thaler, S. L. Bud’ko, P. C. Canfield, A. I. Goldman, I. I. Mazin, R. Prozorov, Uniaxial-strain mechanical detwinning of CaFe_2_As_2_ and BaFe_2_As_2_ crystals: Optical and transport study. Phys. Rev. B 81, 184508 (2010).

[44] H. Simons, A. C. Jakobsen, S. R. Ahl, C. Detlefs, H. F. Poulsen, Multiscale 3D characterization with dark-field x-ray microscopy. MRS Bull. 41, 454–459 (2016).

[45] C. Yildirim, P. Cook, C. Detlefs, H. Simons, H. F. Poulsen, Probing nanoscale structure and strain by dark-field x-ray microscopy. MRS Bull. 45, 277–282 (2020).

[46] H. F. Poulsen, A. C. Jakobsen, H. Simons, S. R. Ahl, P. K. Cook, C. Detlefs, X-ray diffraction microscopy based on refractive optics. J. Appl. Cryst. 50, 1441–1456 (2017).

[47] E. Kisiel, P. Salev, I. Poudyal, D. J. Alspaugh, F. Carneiro, E. Qiu, F. Rodolakis, Z. Zhang, O. G. Shpyrko, M. Rozenberg, I. K. Schuller, Z. Islam, A. Frano, High-resolution full-field structural microscopy of the voltage-induced filament formation in VO_2_-based neuromorphic devices. ACS Nano 19, 15385–15394 (2025). 40227001 10.1021/acsnano.4c14696 PMC12044682

[48] E. Kisiel, I. Poudyal, P. Kenesei, M. Engbretson, A. Last, R. Basak, I. Zaluzhnyy, U. Goteti, R. Dynes, A. Miceli, A. Frano, Z. Islam, Direct detection system for full-field nanoscale X-ray diffraction-contrast imaging. Opt. Express 32, 27682–27689 (2024). 39538600 10.1364/OE.518974

[49] H. Kleinert, “Part III: Gauge fields in solids,” in *Gauge Fields in Condensed Matter* (World Scientific, 1989), vol. 2, pp. 745–1329.

[50] W. J. Meese, R. M. Fernandes, Compatible instability: Gauge constraints of elasticity inherited by electronic nematic criticality. arXiv:2507.23753 [cond-mat.str-el] (2025). 10.1103/wytr-kd9j 42113139

[51] W. J. Meese, R. M. Fernandes, Theory of electronic nematic criticality constrained by elastic compatibility. arXiv:2507.23754 [cond-mat.str-el] (2025).

[52] K. O. Rasmussen, T. Lookman, A. Saxena, A. R. Bishop, R. C. Albers, S. R. Shenoy, Three-dimensional elastic compatibility and varieties of twins in martensites. Phys. Rev. Lett. 87, 055704 (2001). 11497786 10.1103/PhysRevLett.87.055704

[53] R. T. Brierley, P. B. Littlewood, Domain wall fluctuations in ferroelectrics coupled to strain. Phys. Rev. B 89, 184104 (2014).

[54] I. Paul, M. Garst, Lattice effects on nematic quantum criticality in metals. Phys. Rev. Lett. 118, 227601 (2017). 28621984 10.1103/PhysRevLett.118.227601

[55] E. Dagotto, Complexity in strongly correlated electronic systems. Science 309, 257–262 (2005). 16002608 10.1126/science.1107559

[56] K. Lai, M. Nakamura, W. Kundhikanjana, M. Kawasaki, Y. Tokura, M. A. Kelly, Z. X. Shen, Mesoscopic percolating resistance network in a strained manganite thin film. Science 329, 190–193 (2010). 20616272 10.1126/science.1189925

[57] Z. Jin, S. Ismail-Beigi, First-principles prediction of structural distortions in the cuprates and their impact on the electronic structure. Phys. Rev. X 14, 041053 (2024).

[58] D. Gürsoy, K. A. Yay, E. Kisiel, M. Wojcik, D. Sheyfer, A. Last, M. Highland, I. R. Fisher, S. Hruszkewycz, Z. Islam, Dark-field X-ray microscopy with structured illumination for three-dimensional imaging. Commun. Phys. 8, 34 (2025).

[59] A. Lahiri, A. Klein, R. M. Fernandes, Defect-induced electronic smectic state at the surface of nematic materials. Phys. Rev. B 106, L140503 (2022).

[60] M. S. Ikeda, T. Worasaran, E. W. Rosenberg, J. C. Palmstrom, S. A. Kivelson, I. R. Fisher, Elastocaloric signature of nematic fluctuations. Proc. Natl. Acad. Sci. U.S.A. 118, e2105911118 (2021). 34503998 10.1073/pnas.2105911118 PMC8449397

[61] S. Lederer, Y. Schattner, E. Berg, S. A. Kivelson, Enhancement of superconductivity near a nematic quantum critical point. Phys. Rev. Lett. 114, 097001 (2015). 25793842 10.1103/PhysRevLett.114.097001

[62] A. T. Hristov, M. S. Ikeda, J. C. Palmstrom, P. Walmsley, I. R. Fisher, Elastoresistive and elastocaloric anomalies at magnetic and electronic-nematic critical points. Phys. Rev. B 99, 100101 (2019).

[63] J. Garriga Ferrer, R. Rodríguez-Lamas, H. Payno, W. De Nolf, P. Cook, V. A. Solé Jover, C. Yildirim, C. Detlefs, *darfix* – Data analysis for dark-field X-ray microscopy. J. Synchrotron Radiat. 30, 527–537 (2023). 37000183 10.1107/S1600577523001674 PMC10161887

[64] R. Bansal, G. Raj, T. Choudhury, “Blur image detection using Laplacian operator and Open-CV,” in *2016 International Conference System Modeling & Advancement in Research Trends (SMART)* (IEEE, 2017), pp. 63–67.

[65] S. Pertuz, D. Puig, M. A. Garcia, Analysis of focus measure operators for shape-from-focus. Pattern Recognit. 46, 1415–1432 (2013).

[66] R. M. Fernandes, A. V. Chubukov, J. Knolle, I. Eremin, J. Schmalian, Preemptive nematic order, pseudogap, and orbital order in the iron pnictides. Phys. Rev. B 85, 024534 (2012).

[67] W. J. Meese, “Consequences of nematoelasticity in structurally disordered quantum materials,” thesis, University of Minnesota Twin Cities (2024).

[68] J. D. Eshelby, “The Continuum Theory of Lattice Defects,” in *Solid State Physics*, F. Seitz, D. Turnbull, Eds. (Academic Press, 1956), vol. 3, pp. 79–144.

[69] R. deWit, “Linear theory of static dislocations,” in *Fundamental Aspects of Dislocation Theory*, J. A. Simmons, R. deWit, R. Bullough, Eds. (National Bureau of Standards, 1970), vol. 1, pp. 651–673. 10.6028/jres.073A.042 PMC6658428 31929648

[70] R. deWit, Theory of disclinations: II. Continuous and discrete disclinations in anisotropic elasticity. J. Res. Natl. Bur. Stand. A Phys. Chem. 77A, 49–100 (1973). 32189727 10.6028/jres.077A.003 PMC6742835

[71] R. deWit, Theory of disclinations: III. Continuous and discrete disclinations in isotropic elasticity. J. Res. Natl. Bur. Stand. A Phys. Chem. 77A, 359–368 (1973). 32189746 10.6028/jres.077A.024 PMC6715976

[72] R. deWit, Theory of disclinations: IV. Straight disclinations. J. Res. Natl. Bur. Stand. A Phys. Chem. 77A, 607–658 (1973). 32189758 10.6028/jres.077A.036 PMC6728463

[73] E. Kröner, “Continuum theory of defects,” in *Physics of Defects*, Les Houches, Session 35, R. Balian, M. Kléman, J.-P. Poirier, Eds. (North-Holland Pub. Co., Amsterdam, 1981), pp. 215–315.

[74] T. Mura, *Micromechanics of Defects in Solids*, vol. 3 of *Mechanics of Elastic and Inelastic Solids* (Springer, ed. 2, 1987).

[75] R. Gröger, T. Lookman, A. Saxena, Defect-induced incompatibility of elastic strains: Dislocations within the Landau theory of martensitic phase transformations. Phys. Rev. B 78, 184101 (2008).

[76] A. J. Beekman, J. Nissinen, K. Wu, K. Liu, R.-J. Slager, Z. Nussinov, V. Cvetkovic, J. Zaanen, Dual gauge field theory of quantum liquid crystals in two dimensions. Phys. Rep. 683, 1–110 (2017).

[77] M. Pretko, L. Radzihovsky, Fracton-elasticity duality. Phys. Rev. Lett. 120, 195301 (2018). 29799220 10.1103/PhysRevLett.120.195301

[78] M. Pretko, Z. Zhai, L. Radzihovsky, Crystal-to-fracton tensor gauge theory dualities. Phys. Rev. B 100, 134113 (2019).

[79] J. Gaa, G. Palle, R. M. Fernandes, J. Schmalian, Fracton-elasticity duality in twisted moiré superlattices. Phys. Rev. B 104, 064109 (2021).

[80] R. A. Cowley, Acoustic phonon instabilities and structural phase transitions. Phys. Rev. B 13, 4877–4885 (1976).

[81] R. Folk, H. Iro, F. Schwabl, Critical statics of elastic phase transitions. Z. Phys. B: Condens. Matter 25, 69–81 (1976).

[82] U. Karahasanovic, J. Schmalian, Elastic coupling and spin-driven nematicity in iron-based superconductors. Phys. Rev. B 93, 064520 (2016).

[83] R. M. Fernandes, J. W. F. Venderbos, Nematicity with a twist: Rotational symmetry breaking in a moiré superlattice. Sci. Adv. 6, eaba8834 (2020). 32821828 10.1126/sciadv.aba8834 PMC7406379

[84] M. Hecker, R. M. Fernandes, Phonon-induced rotation of the electronic nematic director in superconducting Bi_2_Se_3_. Phys. Rev. B 105, 174504 (2022).

[85] L. D. Landau, E. M. Lifshitz, *Theory of Elasticity*, vol. 7 of *Course of Theoretical Physics* (Pergamon Press, ed. 2, 1970).

