# Supplementary material for: Discovery of spontaneous mesoscopic strain waves in nematic domains using dark-field x-ray microscopy
Source: Sci Adv. 2026 May 20;12(21):eaec8998. doi: 10.1126/sciadv.aec8998 (PMC13189129; doi:10.1126/sciadv.aec8998)
Supplement: Supplementary file 1 — Supplementary Text Figs. S1 to S7 References [file sciadv.aec8998_sm.pdf]

Science Advances
AAAS

Supplementary Materials for

Discovery of spontaneous mesoscopic strain waves in nematic domains using dark-field x-ray microscopy

Kaan A. Yay *et al.*

Corresponding author: Kaan A. Yay, kaanalpyay@stanford.edu; Zahir Islam, zahir@anl.gov; Ian R. Fisher, irfisher@stanford.edu

Sci. Adv. **12**, eaec8998 (2026)
DOI: 10.1126/sciadv.aec8998

This PDF file includes:

Supplementary Text
Figs. S1 to S7
References

Supplementary Text

S1. Reconstruction of DFXM data

Pre-processing and noise removal

All raw images from the Zyla camera in .tiff file format were pre-processed using (1) background subtraction, followed by (2) cropping to the region of interest, (3) threshold removal, and (4) removal of isolated pixels. Most of the 2558×2158 pixels of the camera for a given image did not contain a diffraction signal, therefore we used the median value of the intensity distribution of all pixels as the background value for a given image. This background value remained static between different sets of images and was subtracted from each image. The images were then cropped to an empirically determined region of interest to reduce the computational cost of further analysis. In the cropped image, all pixel values below an empirically determined threshold were set to zero to remove random noise. Finally, isolated pixels with high intensity whose neighbors had zero intensity were also set to zero. Our pre-processing methodology follows steps similar to those of the *darfix* package in Python (*63*).

Selection of images with sharpest features from a particular scan

Our datasets consisted of (1) θ scans where we stepped over individual θ motor values within a designated angular interval at a fixed 2θ value, or (2) θ-2θ scans where we iterated over a nested loop of θ and 2θ motor values. For each $(\theta, 2\theta)$ value in either of these two types of scans, we took multiple images for redundancy. An example dataset of images taken consecutively at a fixed $(\theta, 2\theta)$ value within the 040_1 peak at 80 K can be seen in Fig. S1**b**. As can be seen in this example dataset, some images within the set have sharper features, whereas the others have more blur, which we ascribe to the small yet present vibrations coming from the compressor of the cryostat.

To automatically select the sharpest image from a given dataset, we used a method involving the Laplacian operator of the Open Source Computer Vision (OpenCV) library. The Laplacian operator approximates the second derivative of the pixel intensity with respect to pixel position and is therefore sensitive to detecting sharp edges. Therefore, the variance and the maximum value of the Laplacian of a given image are commonly used metrics in computer vision to quantify the sharpness of that image: the higher the variance or the maximum of the Laplacian, the sharper the

image (*64, 65*).

The algorithm we used to detect the sharpest image from a dataset is as follows:

1. Smoothen each image by convolving it with a Gaussian filter to reduce noise, (using the GaussianBlur function of OpenCV)

2. Apply Laplacian operator to each image and compute the variance and maximum value of the resulting Laplacian images,

3. Normalize the Laplacian variance and maximum values for each image with respect to the difference between their maximum and minimum values within a given dataset. So, for $\mathrm{Var}L(i)$ being the Laplacian variance and $\mathrm{Max}L(i)$ being the maximum value of the Laplacian of image i:

$$\mathrm{Var}L(i)_{\mathrm{norm}} = \frac{\mathrm{Var}L(i) - \min_i(\mathrm{Var}L(i))}{\max_i(\mathrm{Var}L(i)) - \min_i(\mathrm{Var}L(i))}$$

$$\mathrm{Max}L(i)_{\mathrm{norm}} = \frac{\mathrm{Max}L(i) - \min_i(\mathrm{Max}L(i))}{\max_i(\mathrm{Max}L(i)) - \min_i(\mathrm{Max}L(i))}$$

4. Select the sharpest image by determining the image with the highest quadrature sum of normalized Laplacian variance and maximum values:

$$i_{\mathrm{sharpest}} = \mathrm{argmax}_i \left\{ (\mathrm{Var}L(i)_{\mathrm{norm}})^2 + (\mathrm{Max}L(i)_{\mathrm{norm}})^2 \right\}$$

As can be seen in the lowest panel of Fig. S1**a** the normalized Laplacian variance and Laplacian maximum values for images from the example dataset correspond to each other in an approximately linear fashion. Our algorithm amounts to selecting the image with the largest radius in this two-dimensional space of sharpness metrics. A visual comparison of the sharpness metrics of each image in Fig. S1**a** with the actual images in Fig. S1**b** confirms that the used metrics indeed distinguish the sharpest images (20 and 7) from blurrier ones. In this dataset, the algorithm would select image 20 as the sharpest image for further analysis. We empirically confirmed the success of this algorithm in selecting the sharpest images in several other datasets.

Image registration and construction of strain maps from θ–2θ scans

To construct the strain map, we selected images whose $(\theta, 2\theta)$ values lie on a line cut along the axis of the scattering vector $\vec{Q}$ through the investigated Bragg peak. The intensity in these images correspond to diffraction coming from parts of the illuminated area of the sample with different d-spacing (strain) values.

Because our detector is stationary during a θ–2θ scan, a change in 2θ leads to a finite shift of images along both the horizontal and vertical directions of the detector pixel array. Therefore, image registration is necessary to assign pixels from different images to the correct locations on the sample. Our image registration process consisted of (1) determining the (x, y) shift between successive images by calculating their cross-correlation using the 2D discrete Fourier transform (fft2) function of NumPy, and (2) shifting the images according to their determined shift value using the ndimage.shift function of SciPy, which performs shifts with subpixel accuracy using a cubic spline interpolation. We empirically confirmed that the (x, y) shift between different images are linear as a function of 2θ.

After the shift correction and image registration, we assigned each pixel (x, y) its corresponding 2θ value by determining the center of mass of the intensity distribution as a function of 2θ. The corresponding d-spacing value for each pixel was then obtained using Bragg's law, and the relative strain $\Delta\varepsilon_{xy}$ was calculated by finding the relative difference in the d-spacing at each pixel from the median d-spacing value in the imaged region.

$$d = \frac{\lambda}{2 \sin \frac{2\theta}{2}} \tag{S1}$$

$$\Delta\varepsilon_{xy} = \frac{d - d_{\text{med}}}{d + d_{\text{med}}} \approx \frac{d - d_{\text{med}}}{2d_{\text{med}}} \tag{S2}$$

We used the notation $\Delta\varepsilon_{xy} = \Delta\varepsilon_{B_{2g}}$ to describe the variation of the d-spacing of both the (220) lattice planes in the high-temperature tetragonal phase (Fig. 3C) and the $(040)_1$ lattice planes in the orthorhombic phase (Fig. 3A) to emphasize the B_{2g} character of the spontaneously developing electronic nematicity in this material, which is defined with respect to the D_{4h} point group of the parent tetragonal phase.

Construction of large area local orientation maps from θ scans

We constructed the large area maps shown in Fig. 3 by performing a fine θ scan at 100 different locations in the sample (a 10×10 array of (x, y) positional values on the sample). To determine the variation of the diffraction intensity for a given θ value across the whole $\sim 100\ \mu$m $\times$ 100 μm area, we shifted each image in the array by the known shift value of the motor moving the sample. The pixel intensities of adjacent images were averaged for the regions in which they overlapped. An example large area diffraction intensity map for $\theta = 10.086^\circ$ is given in Fig. 3B. The local orientation map for the large area was then calculated by finding the center of mass of the intensity distribution at each individual pixel as a function of θ, and the resulting θ value was assigned to that pixel. The resulting local orientation map is shown in Fig. 3A.

S2. Fourier transform studies

Generation of aggregate Fourier transform plots

Each dataset used in the Fourier transform (FT) study consisted of 10,000–100,000 images taken at a given temperature. The datasets collected at 80 K and 60 K were part of a θ–2θ scan of the 040_1 peak with multiple images taken at each $(\theta, 2\theta)$ value and the sample location investigated was fixed. The dataset collected at 3 K was part of a large area θ scan of the 400_4 peak; a θ scan was performed at several different locations on the sample, and multiple images were taken for each θ value. To construct the Fourier transform plots displayed in Fig. 4C, we first selected a subset of the images taken at every $(\theta, 2\theta)$ value in a given dataset and pre-processed them as described above. Then, we used the fft2 function of NumPy to determine the 2D FT of each image, and added the absolute value of the FTs of all images for every given $(\theta, 2\theta)$ value. The absolute values of all the FTs of all θ values were then added for a particular 2θ value at the center of the corresponding Bragg peak. Finally, the logarithm of the aggregate FT arrays was computed to create the FT plots shown in Fig. 4C. The procedure used to generate the plots was applied to all other 2θ values for the 80 K and 60 K datasets, and to multiple locations on the sample for the 3 K dataset.

Wavelength determination

Once the aggregate FT plots were generated, we determined the average wavelength corresponding to each 2θ value and sample location using an automated procedure. First, to quantify the

angular orientation of satellite peaks in a two-dimensional FT, we developed a method that identifies and analyzes high-intensity features in the FT image. The FT was first filtered using a cross-shaped mask to block the central peak and detector artifacts along the x and y axes, thereby enhancing the visibility of satellite peaks. A dynamic thresholding scheme based on the image's intensity standard deviation was applied to isolate prominent peaks, and connected component labeling was used to identify and retain notable high-intensity clusters. Once two satellite peaks with the highest intensities were isolated, their centers of mass were calculated, and a linear fit was performed to determine their angular deviation from the horizontal axis. The method returned the rotation angle of the peaks. This automated approach ensured robustness against noise and spurious features by adaptively refining the threshold until exactly two dominant satellite peaks were identified.

The spectrum was then rotated according to the determined angle to align the peaks horizontally, and a region of interest was extracted and averaged along the y-axis to yield a one-dimensional intensity profile, as depicted on the right panel of Fig S2.

To extract the real-space wavelength from the one-dimensional Fourier amplitude profile, satellite peaks were identified using the find_peaks method of SciPy, a prominence-based peak-finding algorithm. The prominence threshold was adaptively tuned until exactly three peaks were detected, corresponding to the central and the two main satellite peaks expected from the periodic modulation. A Gaussian fit was then applied to a windowed region around the right satellite peak to determine its center with subpixel precision. The corresponding wavenumber was calculated in spatial frequency units ($\mu\mathrm{m}^{-1}$), and the wavelength was obtained as its inverse in microns. If the satellite peaks could not be reliably detected due to falling out of the Bragg condition at certain 2θ values or locations on the sample, the function returned zero.

The results of the described wavelength determination at differing 2θ values for the datasets collected at 80 K and 60 K, and at differing sample locations for the dataset collected at 3 K, are shown in Fig. S3. We collected the 80 K and 60 K data consecutively at the same location on the sample by performing θ–2θ scans on the same peak 040_1. We determined that the difference between the means of the wavelength values at the two temperatures was not significant, given the variance in the datasets. The dataset at 3 K, however, has a mean wavelength value significantly larger than that of the other datasets. It is important to note that the 3 K dataset was collected by imaging the 400_4 peak, and hence the observed modulations originated from a different domain than the one

imaged at higher temperatures. The domain size itself potentially plays a role in the wavelength of the modulations. Thus, it is difficult to make a one-to-one comparison of the wavelength values measured in this domain versus the other to extract an empirical dependence of the wavelength of modulations on temperature. Nonetheless, the fact that the mean wavelength does not change significantly between 80 K and 60 K and is of the same order of magnitude across the three datasets analyzed suggests that the wavelength of modulations varies weakly with temperature, and that the two different domains investigated are of roughly similar size.

In a related observation, the histograms for 80 K and 60 K show a small relative variation from the mean of the detected wavelength values as 2θ is varied. In contrast, the histogram for 3 K shows a much larger relative variation from the mean value. We ascribe the larger variance in the 3 K dataset to two factors. First, we determined that the 3 K dataset consisted of blurrier images with inherently less sharp features; therefore, the FT analysis resulted in satellite peaks also with broader features, adding extra variance to the wavelength determination. In many images, the blurriness prevented our algorithm from detecting any prominent peaks, and these images were not included in the wavelength analysis. Second, the 3 K dataset was collected for the 400_4 peak at 100 different locations on the sample (a 10×10 array of (x, y) positions), whereas the 80 K and 60 K datasets were collected for the 040_1 peak at a single location. As the 3 K dataset covers a larger area of the investigated domain, it potentially includes effects closer to domain walls or other external strain effects that can introduce added variance to the wavelength of the observed modulations.

Observation of subdominant peaks in the Fourier transform plots

From Fig. 4C we notice that there are additional peaks in the FT plots with smaller intensity besides the dominant satellite peaks. These subdominant peaks are especially visible in the FT plots of the 040_1 peak at 80 K and 60 K as these datasets consist of sharper images compared to the 3 K dataset as discussed above. As the FT plots at 60 K and 80 K in Fig. 4**c** consist of the sum of the FT of multiple images at several different θ values for a given 2θ value, we also checked the FT plots of individual images to confirm that the subdominant peaks are inherent to all images and not merely an artifact of the aggregation procedure discussed above. The resulting FT plots of the individual images displayed in Fig. S1**b** are shown in Fig. S4. As these FT plots demonstrate, the subdominant peaks are present for each image. Examining the FT plots of individual images in Fig. S4 reveals

that that the subdominant peaks are periodic. We ascribe this observation to a combination of two possible causes: (1) The pinhole in front of the objective lens acts as a circular mask on the image, and (2) there is a slight wavelength modulation approximately every five periods. While the cause of the wavelength modulation is currently unknown to us, both of these effects would lead to an FT spectrum with periodic sidebands around the main satellite peaks described by Bessel functions of the first kind, explaining the subdominant peak spectrum we observe.

S3. Nemato-elasticity and shear strain waves

In this section, we use a two-dimensional Ginzburg-Landau formalism to capture the interaction of the strain tensor and the electronic nematic order parameter in iron pnictide $Ba(Fe_{0.98}Cu_{0.02})_2As_2$. We will show that the full nemato-elastic problem in two spatial dimensions with three interacting fields naturally leads to the effective one-dimensional problem described in the main text. We then show that a single sinusoid and a perfectly uniform square wave are both unstable towards the partial square-wave *ansatz* taken in the main text. This holds within the nematic phase, with the solution vanishing completely above the nematic transition temperature. The minimizing partial wave solution for the twin has an accompanying temperature-dependent spatial modulation in the bulk of the twin components. Both the length scale and amplitude of the spatial modulation are slowly decreasing functions of temperature in the ordered phase, each scaling as $1/\sqrt{T^* - T}$.

Development of spontaneous strain waves

The parent compound, $BaFe_2As_2$, is well known to exhibit an Ising electronic nematic instability before the onset of stripe-antiferromagnetic order (*6, 11, 34, 66*). Meanwhile, the development of a nonzero, ferroelastic shear strain is a consequence of the symmetry-induced nemato-elastic bilinear coupling that the Ising nematic order parameter, ϕ, has with the shear strain, ε_{xy}. The full nemato-elastic free energy then is the sum of three parts, and is written as

$$F = F_{\mathrm{n}}\left[\phi\left(\boldsymbol{r}\right)\right] + F_{\mathrm{e}}\left[\varepsilon_{ij}\left(\boldsymbol{r}\right)\right] + F_{\mathrm{ne}}\left[\phi\left(\boldsymbol{r}\right), \varepsilon_{ij}\left(\boldsymbol{r}\right)\right], \tag{S3}$$

where the bare electronic nematic and bare elastic free energies assume the form

$$F_{\mathrm{n}}\left[\phi\left(\boldsymbol{r}\right)\right]=\frac{1}{2}\int_{r}\left\{r\phi^{2}\left(\boldsymbol{r}\right)+c\left[\nabla\phi\left(\boldsymbol{r}\right)\right]^{2}\right\}+u\int_{r}\phi^{4}\left(\boldsymbol{r}\right), \tag{S4}$$

$$F_{\mathrm{e}}\left[\varepsilon_{ij}\left(\boldsymbol{r}\right)\right]=\frac{1}{2}\int_{r}\left\{B\left(\varepsilon_{xx}+\varepsilon_{yy}\right)^{2}+\mu_{1}\left(\varepsilon_{xx}-\varepsilon_{yy}\right)^{2}+\mu_{2}\left(2\varepsilon_{xy}\right)^{2}\right\}. \tag{S5}$$

The nemato-elastic bilinear, meanwhile, is given by

$$F_{\mathrm{ne}}\left[\phi\left(\boldsymbol{r}\right),\varepsilon_{ij}\left(\boldsymbol{r}\right)\right]=-\lambda\int_{r}\phi\left(\boldsymbol{r}\right)\left[2\varepsilon_{xy}\left(\boldsymbol{r}\right)\right]. \tag{S6}$$

In the equations above, we use the shorthand $\int_{r}\equiv\int\mathrm{d}^{2}r$. The quantity $r\propto T-T_{0}^{*}$ tunes through the bare nematic transition temperature, T_{0}^{*}, c is the bare nematic order parameter stiffness, and $u>0$ stabilizes the free energy. The bare elastic free energy is expanded to the harmonic level for the planar tetragonal system, where symmetry allows for three distinct elastic constants, B, μ_1, and μ_2, all of which must be positive for thermodynamic stability. The quantity B is the in-plane bulk modulus, μ_1 is the deviatoric modulus, and μ_2 is the shear modulus. The bulk modulus characterizes the energy scale of symmetry-preserving, volume-changing strains, whereas the deviatoric and shear moduli account for the energy required for symmetry-breaking, volume-preserving strains. The quantity, λ, is the nemato-elastic coupling.

It is more convenient to express the elastic free energy in terms of the following three quantities:

$$\mu_{m}\equiv\frac{\mu_{1}+\mu_{2}}{2},\qquad b\equiv\frac{B}{\mu_{m}},\qquad \delta\mu\equiv\frac{\mu_{1}-\mu_{2}}{\mu_{1}+\mu_{2}}, \tag{S7}$$

where μ_m is the typical energy scale of symmetry-lowering strains, b is a non-universal dimensionless quantity relating the bulk and shear rigidities, and $\delta\mu$ is the dimensionless “tetragonal anisotropy” coefficient. The coefficient, $\delta\mu$, vanishes for two-dimensional hexagonal crystals and isotropic solids. With these quantities, the elastic free energy is

$$F_{\mathrm{e}}\left[\varepsilon_{ij}\left(\boldsymbol{r}\right)\right]=\frac{\mu_{m}}{2}\int_{r}\left\{b\left(\varepsilon_{xx}+\varepsilon_{yy}\right)^{2}+\left(1+\delta\mu\right)\left(\varepsilon_{xx}-\varepsilon_{yy}\right)^{2}+\left(1-\delta\mu\right)\left(2\varepsilon_{xy}\right)^{2}\right\}. \tag{S8}$$

Due to the Saint Venant Compatibility Relations discussed in the main text, and in nemato-elasticity problems within Refs. (*50, 51, 67*), the three strain components of a spatially inhomogeneous deformation are interdependent. This interdependence is the result of the relationship between the strain tensor—the physical quantity which costs elastic energy when nonzero—and the lattice displacement vector. These conditions are inviolate in ideal materials, and their violation in actual

samples is a direct consequence of structural disorder in the form of crystalline defects (*49, 67–79*). Indeed, the fact that the linear strain tensor, ε_{ij}, follows from the displacement vector, $\boldsymbol{u}$, as

$$\varepsilon_{ij}\left(\boldsymbol{r}\right)=\frac{1}{2}\left[\partial_i u_j\left(\boldsymbol{r}\right)+\partial_j u_i\left(\boldsymbol{r}\right)\right], \tag{S9}$$

implies there are only ever two independent strain fields since there are only two components of the displacement vector in two dimensions. Thus, the full nemato-elastic problem in 2D involves *three* independent degrees of freedom: one Ising nematic order parameter, and two independent strain components. To proceed, it is convenient to use a "helical basis" for the strain fields that emphasizes the symmetry and intrinsic directionality of the compatibility relations (*50, 51*). By doing so, one arrives at an elastic free energy in the form

$$F_{\mathrm{e}}\left[\varepsilon_{ij}\left(\boldsymbol{r}\right)\right]=\frac{1}{2V}\sum_{\boldsymbol{q}}\boldsymbol{\varepsilon}_h^{\dagger}\cdot\mathfrak{C}\left(\hat{q}\right)\cdot\boldsymbol{\varepsilon}_h, \tag{S10}$$

where $\boldsymbol{\varepsilon}_h\equiv(\varepsilon_1^h\ \varepsilon_2^h)^{\mathrm{T}}$ is a vector of the Fourier amplitudes of the longitudinal (ε_1^h) and transverse strain components (ε_2^h). The terms "longitudinal" and "transverse" are defined in this context with respect to the wave vector, $\boldsymbol{q}$, and, following Refs. (*50, 51*), are given in terms of the displacement vector, $\boldsymbol{u}$, as

$$\begin{aligned}\varepsilon_1^h&\equiv\mathrm{i}\boldsymbol{q}\cdot\boldsymbol{u},\\ \varepsilon_2^h&\equiv\mathrm{i}\hat{z}\cdot\left(\boldsymbol{q}\times\boldsymbol{u}\right).\end{aligned} \tag{S11}$$

The elastic stiffness matrix, written in the helical basis, is given by

$$\mathfrak{C}\left(\vec{q}\right)=\mu_m\left\{b\boldsymbol{Q}_{A_{1g}}\boldsymbol{Q}_{A_{1g}}^{\mathrm{T}}+\left(1+\delta\mu\right)\boldsymbol{Q}_{B_{1g}}\boldsymbol{Q}_{B_{1g}}^{\mathrm{T}}+\left(1-\delta\mu\right)\boldsymbol{Q}_{B_{2g}}\boldsymbol{Q}_{B_{2g}}^{\mathrm{T}}\right\}, \tag{S12}$$

with the two-component, momentum-dependent form factors being given by

$$\boldsymbol{Q}_{A_{1g}}=\begin{pmatrix}1\\0\end{pmatrix},\quad \boldsymbol{Q}_{B_{1g}}=\begin{pmatrix}\cos 2\zeta\\-\sin 2\zeta\end{pmatrix},\quad \boldsymbol{Q}_{B_{2g}}=\begin{pmatrix}\sin 2\zeta\\\cos 2\zeta\end{pmatrix}. \tag{S13}$$

In the above, the momentum is defined in polar coordinates by $\boldsymbol{q}\equiv(q_x,q_y)\equiv q(\cos\zeta,\sin\zeta)$. These form factors transform the longitudinal, ε_1^h, and the transverse, ε_2^h, strains into the irreducible representations of the strain tensor of tetragonal point group, D_{4h}:

$$\begin{aligned}\varepsilon_{A_{1g}}&\equiv\varepsilon_{xx}+\varepsilon_{yy}=\boldsymbol{Q}_{A_{1g}}^{\mathrm{T}}\cdot\boldsymbol{\varepsilon}_h\\ \varepsilon_{B_{1g}}&\equiv\varepsilon_{xx}-\varepsilon_{yy}=\boldsymbol{Q}_{B_{1g}}^{\mathrm{T}}\cdot\boldsymbol{\varepsilon}_h\\ \varepsilon_{B_{2g}}&\equiv 2\varepsilon_{xy}=\boldsymbol{Q}_{B_{2g}}^{\mathrm{T}}\cdot\boldsymbol{\varepsilon}_h.\end{aligned} \tag{S14}$$

Comparing Eqs. (S13) and (S14), it is clear that the compatibility relations mix the symmetry-breaking strains, $\varepsilon_{B_{1g}}$ and $\varepsilon_{B_{2g}}$, with the symmetry-preserving dilatation strain $\varepsilon_{A_{1g}} = \varepsilon_1^h$. This will generally lead to a nonlocal, long-ranged coupling between the local electronic nematic order parameter and the local strain tensor. Generally, therefore, there is no direct proportionality between the *local* nematic order parameter and the *local* symmetry-breaking shear. This nonlocal relationship exists within bare elasticity theory, and even in the isotropic medium (see Sec. S4 and Refs. (*50,51*)). The nonlocality is present unless either $\cos 2\zeta = 0$ or $\sin 2\zeta = 0$, respectively. In the latter case, since $\sin 2\zeta$ vanishes along the tetragonal coordinate axes, $[100]_T$ and $[010]_T$, we will be able to establish a local proportionality between the shear strain and the nematic order parameter.

One proceeds by minimizing the total free energy with respect to the two strain components, yielding the following coupled equations of state,

$$\mathfrak{C}\left(\hat{q}\right) \cdot \boldsymbol{\varepsilon}_h = \lambda \phi \boldsymbol{Q}_{B_{2g}}. \tag{S15}$$

Along the crystal axes, $\sin 2\zeta = 0$ and $\cos 2\zeta = \pm 1$, in which case these equations of state decouple as

$$\begin{aligned} \mu_m(1 + b + \delta\mu)\varepsilon_1^h &= 0, \\ 2\mu_m(1 - \delta\mu)\varepsilon_{xy} &= \lambda\phi, \end{aligned} \tag{S16}$$

since $2\varepsilon_{xy} = \pm\varepsilon_2^h$ in these directions. In real-space, these expressions yield that the shear strain, as a function of position, is given by $\varepsilon_{xy}(\boldsymbol{r}) \propto \phi(\boldsymbol{r})$. The fact that the direct proportionality exists between the induced shear strain and the electronic nematic order for these high-symmetry directions is a manifestation of "direction-selective criticality" known from ferroelastic (*80, 81*) and electronic nematic (*54, 82–84*) phase transitions, and is ultimately a result of the compatibility relations (SVCR) (*50, 51, 67*). Assuming, for simplicity, that spatial modulations occur exclusively along $[100]_T$, then, after substituting in the strain fields into the free energy, we obtain an effective one-dimensional nematic free energy of the form

$$F_{\text{eff}}\left[\phi\left(\boldsymbol{r}\right)\right] = \frac{1}{2}\int_x \left\{\left(r - \frac{\lambda^2}{\mu_2}\right)\phi^2\left(x\right) + c\left[\partial_x\phi\left(x\right)\right]^2\right\} + u\int_x \phi^4\left(x\right), \tag{S17}$$

which is the free energy quoted in the main text when we define the energy units such that $c^2 = 1$ and set $r - \lambda^2/\mu_2 \equiv a(T - T^*)$, where T^* is the effective transition temperature. One observes that for these modulations, the effective theory no longer depends on the non-universal parameter

$b = 2B/(\mu_1 + \mu_2)$. Because of the direct local proportionality between $\varepsilon_{xy}(x)$ and $\phi(x)$ along $[100]_T$ (and $[010]_T$), the spontaneous development of strain is therefore expressed through the spontaneous development of electronic nematic order which occurs when $r = \lambda^2/\mu_2$.

Partial square wave *ansatz* as a model of twin formation

As discussed in the main text, the mesoscale spatial modulations are observed within individual twin components. Since the boundaries of the twin components are not directly observable in the experiment, we have simplified the problem of twin formation to that of an over-constrained minimization of the following one-dimensional Ginzburg-Landau free energy:

$$F_{\text{eff}}\left[\phi\left(x\right)\right] = \frac{1}{2}\int_0^L \mathrm{d}x\,\left\{a(T-T^*)\phi^2\left(x\right) + \left[\partial_x\phi\left(x\right)\right]^2\right\} + u\int_0^L \mathrm{d}x\,\phi^4\left(x\right). \tag{S18}$$

The equation above appears in the main text, where the spatially modulated B_{2g} electronic nematic order parameter is $\phi(x)$. The quantity $a(T-T^*)$ is the inverse (renormalized) nematic susceptibility with the nemato-elastic transition temperature being T^* enhanced from $T_0^* < T^*$ by elastic fluctuations. The parameter $a > 0$. The quartic coefficient, u, is strictly positive for thermodynamic stability.

Given that the modulations occur along the $[100]_T$-direction, the SVCR decouple $\phi(x)$ from nonlocal dilatation strains, allowing for a local proportionality between the electronic nematic order parameter and the shear strain: $\phi(x) \propto \varepsilon_{xy}(x)$. While the development of the strain in this material is due to electronic nematicity, the *local* proportionality between it and the spatially-modulated shear strain would allow for a similar free energy written entirely in terms of $\varepsilon_{xy}(x)$. This suggests a broader universality for strain waves applying beyond electronic nematicity in the iron pnictides. It is important, however, to recall for arbitrary modulations with off-axis momenta, that this universality would not exist.

For twin formation to stabilize Eq. (S18) on the interval $x \in (0, L)$, one must constrain the nematic order parameter to be nonzero within either twin component, but take on opposite signs. This implies that $\phi(x = L/2) = 0$, if we assume the twin is symmetric. We are free to assume that $\phi(x \in (0, L/2)) > 0$, which implies that $\phi(x \in (L/2, L)) < 0$. However, elasticity requires that the traction on each boundary with unit normal $\boldsymbol{n}$ vanish such that $\sigma_{ij}n_j = 0$, where σ_{ij} is the stress tensor (*74, 85*). In the absence of ε_{xx} or ε_{yy} uniaxial strains, the traction-free boundary condition

reduces to a vanishing shear: $\varepsilon_{xy}(x = 0) = \varepsilon_{xy}(x = L) = 0$. These constitute five boundary conditions for Eq. (S18). However, minimizing the functional yields a one-dimensional nonlinear Helmholtz equation whose solution is only uniquely determined by *two* boundary conditions, not five. Thus, the problem of twin formation is over-constrained, and we instead employ a variational approach. The *ansatz* chosen is the partial square-wave, shown in the main text, and written as

$$\phi_M(s) \equiv \Phi\,\mathcal{S}_M(s) \equiv \Phi \cdot \frac{4}{\pi} \sum_{m\ \mathrm{odd}}^{M} \frac{\sin(2\pi m s)}{m}, \quad s \equiv \frac{x}{L} \in [0, 1], \tag{S19}$$

with cutoff integer M. This cutoff interpolates between a single sinuoidal waveform for $M = 1$ and perfectly flat twin components in the limit $M \to \infty$. In both extremes, there are no additional modulations within either twin component, however, they appear for all intermediate, finite values of $M \geq 3$, as shown in Fig. S5. The amplitude Φ controls the magnitude of the uniform strain component in the twin.

Substituting in the *ansatz* in Eq. (S19) yields a free energy density of the form

$$f(\Phi, M) \equiv \frac{F[\phi_M(x)]}{L} = \frac{1}{2}|r|\tilde{r}(M)\Phi^2 + u\tilde{u}(M)\Phi^4, \tag{S20}$$

which depends only on the variational parameters, Φ, and M. The quantities, $\tilde{r}(M)$ and $\tilde{u}(M)$ are determined by

$$\begin{aligned} \tilde{r}(M) &\equiv \int_0^1 \mathrm{d}s \left\{ \mathrm{sgn}(r)\mathcal{S}_M^2(s) + (Q_0\xi_{\mathrm{MF}})^2 \left[\frac{\partial_s \mathcal{S}_M(s)}{2\pi}\right]^2 \right\}, \\ \tilde{u}(M) &\equiv \int_0^1 \mathrm{d}s\, \mathcal{S}_M^4(s), \end{aligned} \tag{S21}$$

where $\xi_{\mathrm{MF}} \equiv 1/\sqrt{a|T - T^*|}$ and $Q_0 \equiv 2\pi/L$ is the fundamental harmonic. In the usual case of the Φ^4-model with a uniform order parameter—one that cannot satisfy the requisite boundary conditions for twin formation—one replaces $\tilde{r}(M)$ and $\tilde{u}(M)$ with $\mathrm{sgn}(r)$ and 1, respectively. The parameter, $Q_0\xi_{\mathrm{MF}}$, is written explicitly as

$$Q_0\xi_{\mathrm{MF}} = \frac{2\pi\xi_{\mathrm{MF}}}{L} = \frac{2\pi}{L\sqrt{a|T - T^*|}}, \tag{S22}$$

and parametrizes the temperature T in the ordered phase.

Minimizing with respect to the amplitude Φ yields

$$\Phi_\star^2 = -\frac{|r|\tilde{r}(M)}{4u\tilde{u}(M)},$$
$$f(\Phi_\star, M) = \begin{cases} 0, & r \propto T - T^* > 0 \\ -\frac{r^2\tilde{r}^2(M)}{16u\tilde{u}(M)}, & r \propto T - T^* < 0 \end{cases} \tag{S23}$$

Minimizing field configurations, $\phi_{M_\star}(x)$, are shown in Fig. S5 for various temperatures, parameterized through the quantity $Q_0\xi_{\mathrm{MF}} = 2\pi\xi_{\mathrm{MF}}/L \propto 1/\sqrt{T^* - T}$.

From the expressions above, it is clear that the partial square wave *ansatz* is only nonzero in the nematic phase with $T < T^*$. Comparing with the minimizers for a uniform order parameter, one can write the conditions above more succinctly as

$$\Phi_\star^2 = \mathrm{sgn}(r)\left[\frac{\tilde{r}(M_\star)}{\tilde{u}(M_\star)}\right]\Phi_{0,\star}^2,$$
$$f(\Phi_\star, M) = -\frac{\tilde{r}^2(M)}{\tilde{u}(M)}\,|f_0|, \tag{S24}$$

where $\Phi_{0,\star}^2 \equiv -r/4u$ is the minimizing amplitude for the uniform case, and f_0 is the corresponding minimum free energy density. For $T > T^*$, $f_0 = 0$ and for $T < T^*$, $f_0 = -r^2/16u$. By varying the odd integer M at various temperatures, one observes a minimum in the free energy develop for $M > 1$, with the minimizing value of M, denoted as $M_\star$, as shown in Fig. S6(**a**). This shows that uniformity within either twin component is unstable towards additional spatial modulations on top of a bulk background.

We quantify the length scale associated with these spatial modulations through a quantity, Λ_M, which is the distance between the extrema immediately adjacent to the center of either twin component, as shown in the inset of Fig. S5. One can obtain a closed form expression for $\partial_s\,\mathcal{S}_M(s)$ by summing a partial geometric series to obtain

$$\partial_s\,\mathcal{S}_M(s) = \partial_s S_M(s) = \frac{4}{\pi}\cdot 2\pi\left\{\frac{\sin\left[2\pi(M+1)s\right]}{2\sin(2\pi s)}\right\}, \tag{S25}$$

from which it follows that the bulk extrema occur when $2\pi(M+1)x/L = p\pi$ for $p \in \mathbb{Z}^+$. As shown in the inset of Fig. S5, the center of the twin domain is $x/L = 1/4$, which then corresponds to the integer $N \equiv (M+1)/2$. Since M is odd, then N represents the number of terms included in $\mathcal{S}_M(s)$. In the inset, we mark $x_N \equiv L/4$. The adjacent extrema therefore correspond to $x_{N\pm1}$. The spatial

modulation scale, Λ_M follows as

$$\Lambda_M \equiv x_{N+1} - x_{N-1} = L\left\{\frac{N+1}{2(M+1)} - \frac{N-1}{2(M+1)}\right\} = \frac{L}{M+1}. \tag{S26}$$

The value of this length scale evaluated for the minimizing cutoff, $\Lambda_{M_\star}$, is shown in Fig. S6**(b)**, as a function of $1/Q_0\xi_{\text{MF}} = L/2\pi\xi_{\text{MF}}$. As the temperature decreases from nematic criticality, one finds that $M_\star \sim L/\xi_{\text{MF}} \propto \sqrt{T^* - T}$, showing that the modulation scale, $\Lambda_{M_\star}$, is a slowly decreasing function of the temperature: $\Lambda_{M_\star} \sim 1/\sqrt{T^* - T}$.

Similarly, by evaluating the *ansatz* at x_{N+1} and x_N, one can determine the amplitude of the spatial modulations within the twin domains as

$$\delta\phi_{M_\star}(x_N) \equiv \frac{1}{2}\left|\phi_{M_\star}(x_{N+1}) - \phi_{M_\star}(x_N)\right|. \tag{S27}$$

This bulk modulation amplitude is plotted in Fig. S7 as a function of temperature. Far from nematic criticality, it is observed that the amplitude is also a slowly decreasing function of the temperature as well, $\delta\phi_{M_\star} \sim 1/\sqrt{T^* - T}$, just as the bulk modulation scale, $\Lambda_{M_\star}$.

S4. Visualizing elastic strain waves

In this section, we demonstrate how to compute the two-dimensional displacement vector, $\boldsymbol{u}(\boldsymbol{r})$, from a known spatially modulated strain field. In doing so, the strain wave necessarily satisfies the Saint-Venant compatibility relations, and from the displacement vector one can compute the three components of the strain tensor: the dilatation strain $\varepsilon_{xx} + \varepsilon_{yy}$, the deviatoric strain $\varepsilon_{xx} - \varepsilon_{yy}$, and the shear strain ε_{xy}. We will show that generally there is a nonlocal relationship between the spatially modulated strain tensor strictly enforced by the compatibility relations (*50, 51*). However, we show that if the modulations have specific momentum directions, then the nonlocal relationship collapses to a local direct proportionality. We will then simplify the analysis to the case of strain waves with a single well-defined wave vector, and use these results to create Fig. 5 in the main text. To keep the discussion regarding the geometry universal, we refrain in this section from discussing the mechanism for a strain wave, instead focusing on the impact the resulting deformation has on a medium should the wave exist.

We start by assuming the helical strain amplitudes from Eq. (S11) are known functions of the wave vector $\boldsymbol{q}$, $\boldsymbol{\varepsilon}_h = \boldsymbol{\varepsilon}_h(\boldsymbol{q})$. We will simplify down to a single static shear wave after obtaining the

general solution. By inverting Eq. (S11), it follows that the displacement vector amplitudes are

$$\begin{bmatrix} u_x(\boldsymbol{q}) \\ u_y(\boldsymbol{q}) \end{bmatrix} = -\frac{\mathrm{i}}{q} \begin{bmatrix} \cos(\zeta) & -\sin(\zeta) \\ \sin(\zeta) & \cos(\zeta) \end{bmatrix} \begin{bmatrix} \varepsilon_1^h(\boldsymbol{q}) \\ \varepsilon_2^h(\boldsymbol{q}) \end{bmatrix} \equiv -\frac{\mathrm{i}}{q} \mathcal{R}(\hat{q}) \cdot \boldsymbol{\varepsilon}_h(\boldsymbol{q}), \tag{S28}$$

with the wave vector being parameterized by $\boldsymbol{q} \equiv q(\cos(\zeta), \sin(\zeta))$. Inverting the Fourier transform, one obtains the general solution for displacement vector in real-space as

$$\boldsymbol{u}(\boldsymbol{r}) = -\begin{bmatrix} \partial_x & -\partial_y \\ \partial_y & \partial_x \end{bmatrix} \cdot \boldsymbol{W}(\boldsymbol{r}), \tag{S29}$$

where the vector, $\boldsymbol{W}(\boldsymbol{r})$, is defined by

$$\boldsymbol{W}(\boldsymbol{r}) \equiv \int \mathrm{d}^2 r' \, g(\boldsymbol{r} - \boldsymbol{r}') \boldsymbol{\varepsilon}_h(\boldsymbol{r}'), \tag{S30}$$

and corresponds to the nonlocal propagation of helical elastic strain throughout the medium. In calculating Eq. (S29), we have used $\boldsymbol{\varepsilon}_h(\boldsymbol{r}) \equiv \frac{1}{V} \sum_{\boldsymbol{q}} \mathrm{e}^{\mathrm{i}\boldsymbol{q}\cdot\boldsymbol{r}} \boldsymbol{\varepsilon}_h(\boldsymbol{q})$, and in $\boldsymbol{W}$, the function $g(\boldsymbol{r})$ is the Green's function for the Poisson equation in 2D: $g(\boldsymbol{r}) = -\frac{1}{2\pi} \log(r)$ (*51*). This integration is based on the compatibility relations using the helical strain formalism, and produces the lattice displacement vector $\boldsymbol{u}(\boldsymbol{r})$ from a compatible helical strain field uniquely, up to a global *uniform* translation and rotation. Each unit cell, represented schematically in Fig. 5 of the main text as a circle and initially positioned at $\boldsymbol{r}$, is displaced according to the deformation: $\boldsymbol{r} \longrightarrow \boldsymbol{r} + \boldsymbol{u}(\boldsymbol{r})$. This displacement vector, being the integrated *strain*, rather than integrated *stress*, is the solution in any crystal with any point group rotational symmetry.

From Eq. (S29), the dilatation and shear strains can be computed as follows

$$\begin{aligned} \varepsilon_{A_{1g}}(\boldsymbol{r}) &= \boldsymbol{\nabla} \cdot \boldsymbol{u}(\boldsymbol{r}) = \varepsilon_1^h(\boldsymbol{r}), \\ \varepsilon_{x^2-y^2}(\boldsymbol{r}) &= \partial_x u_x(\boldsymbol{r}) - \partial_y u_y(\boldsymbol{r}) = -\left(\partial_x^2 - \partial_y^2\right) W_1(\boldsymbol{r}) + \left(2\partial_x\partial_y\right) W_2(\boldsymbol{r}), \\ \varepsilon_{xy}(\boldsymbol{r}) &= \frac{1}{2}\left[\partial_x u_y(\boldsymbol{r}) + \partial_y u_x(\boldsymbol{r})\right] = -\left(2\partial_x\partial_y\right) W_1(\boldsymbol{r}) - \left(\partial_x^2 - \partial_y^2\right) W_2(\boldsymbol{r}). \end{aligned} \tag{S31}$$

These are general expressions for any 2D crystalline point group even though we have adopted the A_{1g} label for the dilatation from the tetragonal point group D_{4h}. In deriving the dilatation above, we exploited the Green's function property that $\nabla^2 g(\boldsymbol{r}) = -\delta(\boldsymbol{r})$. The second and third lines emphasize the generally nonlocal interdependence between the dilatation, deviatoric, and shear strain components that exists for any crystal symmetry – and persists even in the isotropic

continuum (*50, 51*). This nonlocal interdependence is present for generic spatial modulations, *e.g.* when both partial derivatives are nonzero. To show this interdependence explicitly, we apply to the second (third) line of Eq. (S31) the operator $\partial_x^2 - \partial_y^2$ ($4\partial_x^2\partial_y^2$), and sum the results to find

$$\begin{aligned}(\partial_x^2 - \partial_y^2)\varepsilon_{x^2-y^2}(\boldsymbol{r}) + (2\partial_x\partial_y)(2\varepsilon_{xy}(\boldsymbol{r})) &= -\left[(\partial_x^2 - \partial_x^2)^2 + (2\partial_x\partial_y)^2\right] W_1(\boldsymbol{r}) \\ &= -\nabla^4 W_1(\boldsymbol{r}). \end{aligned} \tag{S32}$$

Using $\nabla^2 g(\boldsymbol{r}) = -\delta(\boldsymbol{r})$ again, it follows that $\nabla^2 W_1(\boldsymbol{r}) = -\varepsilon_1^h(\boldsymbol{r})$. Thus, we recover

$$(\partial_x^2 - \partial_y^2)\varepsilon_{x^2-y^2}(\boldsymbol{r}) + (2\partial_x\partial_y)(2\varepsilon_{xy}(\boldsymbol{r})) = (\partial_x^2 + \partial_y^2)\varepsilon_{A_{1g}}(\boldsymbol{r}), \tag{S33}$$

the exact Saint Venant compatibility relation quoted in the main text. Relabeling $\boldsymbol{r}$ as $\boldsymbol{r}'$, multiplying both sides by $g(\boldsymbol{r} - \boldsymbol{r}')$, and integrating $\boldsymbol{r}'$ over the infinite volume, we recover

$$\varepsilon_{A_{1g}}(\boldsymbol{r}) = -(\partial_x^2 - \partial_y^2) \int \mathrm{d}^2 r'\, g(\boldsymbol{r} - \boldsymbol{r}')\varepsilon_{x^2-y^2}(\boldsymbol{r}') - (2\partial_x\partial_y) \int \mathrm{d}^2 r'\, g(\boldsymbol{r} - \boldsymbol{r}')[2\varepsilon_{xy}(\boldsymbol{r}')]. \tag{S34}$$

To obtain the above result, we integrated by parts assuming that the strains vanish at infinity, and have used the derivative identity $\partial_{j'} g(\boldsymbol{r} - \boldsymbol{r}') = -\partial_j g(\boldsymbol{r} - \boldsymbol{r}')$ to bring the partial derivatives outside of the integrals.

The manipulations above show that there is a nonlocal, modulation-direction-dependent relationship between the three components of the strain tensor in 2D. If we assert that there is only one symmetry-breaking strain wave in the system, and choose it as the shear wave over the deviatoric wave, then the dilatation strain reduces to

$$\varepsilon_{A_{1g}}(\boldsymbol{r}) = -(2\partial_x\partial_y) \int \mathrm{d}^2 r'\, g(\boldsymbol{r} - \boldsymbol{r}')[2\varepsilon_{xy}(\boldsymbol{r}')], \tag{S35}$$

and establishes that the degree of dilatation strain is controlled by partial differentiation – thus the direction of spatial modulations control the interdependence between the dilatation and the shear strains. If there is a shear wave that only depends on the $\hat{x} = [100]$ direction, namely $\partial_y \varepsilon_{xy}(\boldsymbol{r}) = 0$ and $\partial_x \varepsilon_{xy}(\boldsymbol{r}) \neq 0$, then it follows from the Green's function that the convolution only depends on x as well. After partial differentiation, the dilatation vanishes, showing that the shear wave can grow arbitrarily large without inducing dilatation strains. Alternatively, taking the modulations in the shear wave to be only along the [110]-direction, such that $(\partial_x - \partial_y)\varepsilon_{xy}(\boldsymbol{r}) = 0$ while $(\partial_x + \partial_y)\varepsilon_{xy}(\boldsymbol{r}) \neq 0$, then it can be shown from Eq. (S35) that the dilatation and shear strains are directly proportional: $\varepsilon_{A_{1g}}(x + y) \propto \varepsilon_{xy}(x + y)$.

We now focus on the case of static waves with well-defined wave vector. Returning to the helical strain basis as the independent degrees of freedom, we seek the displacement vector and the three strain components that the helical strain waves induce. Assume that the helical strain is a static wave in real-space, such that

$$\boldsymbol{\varepsilon}_h(\boldsymbol{r}) \equiv \boldsymbol{\varepsilon}_0 \cos(\boldsymbol{Q} \cdot \boldsymbol{r}), \tag{S36}$$

where $\boldsymbol{\varepsilon}_0$ is a constant two-component vector. The helical strain amplitude follows from Fourier transformation as

$$\boldsymbol{\varepsilon}_h(\boldsymbol{q}) = \frac{1}{2} V \boldsymbol{\varepsilon}_0 \left(\delta_{\boldsymbol{q},\boldsymbol{Q}} + \delta_{\boldsymbol{q},-\boldsymbol{Q}}\right), \tag{S37}$$

where $V \equiv L_x L_y$ is the macroscopic area of the 2D system. Substituting the above into Eq. (S29) yields

$$\boldsymbol{u}(\boldsymbol{r}) = \frac{1}{2\mathrm{i}Q}\left[\mathrm{e}^{\mathrm{i}\boldsymbol{Q}\cdot\boldsymbol{r}} \mathcal{R}(\hat{Q}) + \mathrm{e}^{-\mathrm{i}\boldsymbol{Q}\cdot\boldsymbol{r}} \mathcal{R}(-\hat{Q})\right] \cdot \boldsymbol{\varepsilon}_0 = \frac{\sin(\boldsymbol{Q}\cdot\boldsymbol{r})}{Q} \begin{bmatrix} \varepsilon_{0,1}\cos(\theta_Q) - \varepsilon_{0,2}\sin(\theta_Q) \\ \varepsilon_{0,1}\sin(\theta_Q) + \varepsilon_{0,2}\cos(\theta_Q) \end{bmatrix}. \tag{S38}$$

where $\boldsymbol{Q} \equiv Q(\cos(\theta_Q), \sin(\theta_Q))$ and $\mathcal{R}(-\hat{Q}) = -\mathcal{R}(\hat{Q})$. The dilatation, deviatoric, and shear waves follow as

$$\begin{aligned} \varepsilon_{A_{1g}}(\boldsymbol{r}) &= \varepsilon_{0,1}\cos(\boldsymbol{Q}\cdot\boldsymbol{r}), \\ \varepsilon_{x^2-y^2}(\boldsymbol{r}) &= \left[\varepsilon_{0,1}\cos(2\theta_Q) - \varepsilon_{0,2}\sin(2\theta_Q)\right]\cos(\boldsymbol{Q}\cdot\boldsymbol{r}), \\ \varepsilon_{xy}(\boldsymbol{r}) &= \frac{1}{2}\left[\varepsilon_{0,1}\sin(2\theta_Q) + \varepsilon_{0,2}\cos(2\theta_Q)\right]\cos(\boldsymbol{Q}\cdot\boldsymbol{r}). \end{aligned} \tag{S39}$$

When there is only one symmetry-breaking strain wave in system, the expressions above further simplifies. Consider the case that between the deviatoric and shear strains, only the shear strain is nonzero. Then it follows that

$$\varepsilon_{x^2-y^2}(\boldsymbol{r}) = 0 \quad \Rightarrow \quad \varepsilon_{0,1}\cos(2\theta_Q) = \varepsilon_{0,2}\sin(2\theta_Q). \tag{S40}$$

We see from the above that if $\sin(2\theta_Q) = 0$, then the dilatation amplitude, $\varepsilon_{0,1}$ must vanish. This happens when the wave vector lies along the [100] and [010] axes. Thus, not only is the shear strain independent of the dilatation, without the deviatoric strain, the dilatation strain is exactly zero. Likewise, if $\cos(2\theta_Q) = 0$, then the transverse amplitude $\varepsilon_{0,2}$ must vanish. In this situation, which occurs along the [110] and [$1\bar{1}0$] axes, the opposite is true. Now the shear wave generates a displacement vector that maximizes the magnitude of the dilatation strain. For any other direction

of momentum, it follows that $\varepsilon_{0,2} = \varepsilon_{0,1}\cot(2\theta_Q)$. Substituting this into the expression for the shear strain and simplifying yields

$$\varepsilon_{A_{1g}}(\boldsymbol{r}) = \left[\sin(2\theta_Q)\right]\, 2\varepsilon_{xy}(\boldsymbol{r}), \tag{S41}$$

showing that the amount of dilatation strain accompanying a shear wave is indeed directly controlled by the momentum direction, θ_Q. This expression is equivalent to the Saint Venant compatibility relation in Eq. (S35), applied to the case of a shear wave with vanishing deviatoric strain. In the limit that the wave vector lies along the [100] or [010] axes, then $\varepsilon_{A_{1g}}(\boldsymbol{r}) = 0$, regardless of the amplitude associated with the shear strain wave.

Fig. 5 of the main text illustrates how the degree of interdependence of the shear and the dilatation waves is controlled by the modulation direction, θ_Q. The former is softened near the tetragonal-to-orthorhombic phase transition, whereas the latter is not since the symmetry-preserving dilatation remains gapped at the transition (*50, 51, 80, 82*). To produce the figure, we use the wave vector direction in Eq. (S39) and Eq. (S40) to isolate the shear wave from the dilatation wave in Fig. 5(A, C) with $\theta_Q = 0$, and maximize the dilatation in Fig. 5(B, D) with $\theta_Q = \pi/4$. We then use Eq. (S38) to displace each unit cell by the appropriate local displacement vector. Starting with $\theta_Q = 0$, it follows that $\sin(2\theta_Q) = 0$, and Eq. (S40) restricts the only nonzero solution for the strain wave to have helical amplitudes $\boldsymbol{\varepsilon}_0 \equiv (0, \varepsilon_0)^{\mathrm{T}}$. The dilatation and shear strains, as well as the displacement vector, are then

$$\begin{aligned} \varepsilon_{A_{1g}}(x,y) &= 0, & u_x(x,y) &= 0, \\ \varepsilon_{xy}(x,y) &= \frac{1}{2}\varepsilon_0\cos(Qx), & u_y(x,y) &= \frac{\varepsilon_0}{Q}\sin(Qx), \end{aligned} \tag{S42}$$

which correspond to Fig. 5**(a,c)**. This shows that for $\hat{Q} \propto [100]$, the symmetry-breaking shear strain amplitude, ε_0, can grow arbitrarily large without inducing costly volume-changing dilatation. This is not generally true. For example, changing the direction to $\theta_Q = \pi/4$ such that $\cos(2\theta_Q) = 0$, then Eq. (S40) now restricts $\boldsymbol{\varepsilon}_0 = (\varepsilon_0, 0)^{\mathrm{T}}$. The strains and displacement vector then follow as

$$\begin{aligned} \varepsilon_{A_{1g}}(x,y) &= \varepsilon_0\cos\left[\frac{Q(x+y)}{\sqrt{2}}\right], & u_x(x,y) &= \frac{\varepsilon_0}{Q\sqrt{2}}\sin\left[\frac{Q(x+y)}{\sqrt{2}}\right], \\ \varepsilon_{xy}(x,y) &= \frac{1}{2}\varepsilon_0\cos\left[\frac{Q(x+y)}{\sqrt{2}}\right], & u_y(x,y) &= \frac{\varepsilon_0}{Q\sqrt{2}}\sin\left[\frac{Q(x+y)}{\sqrt{2}}\right], \end{aligned} \tag{S43}$$

These equations correspond to the strain and displacement wave in Fig. 5(B, D). Clearly, a compatible shear wave of any amplitude, ε_0, induces a simultaneous dilatation wave which incurs a higher energy cost if its wave vector satisfies $\cos(2\theta_Q) = 0$.

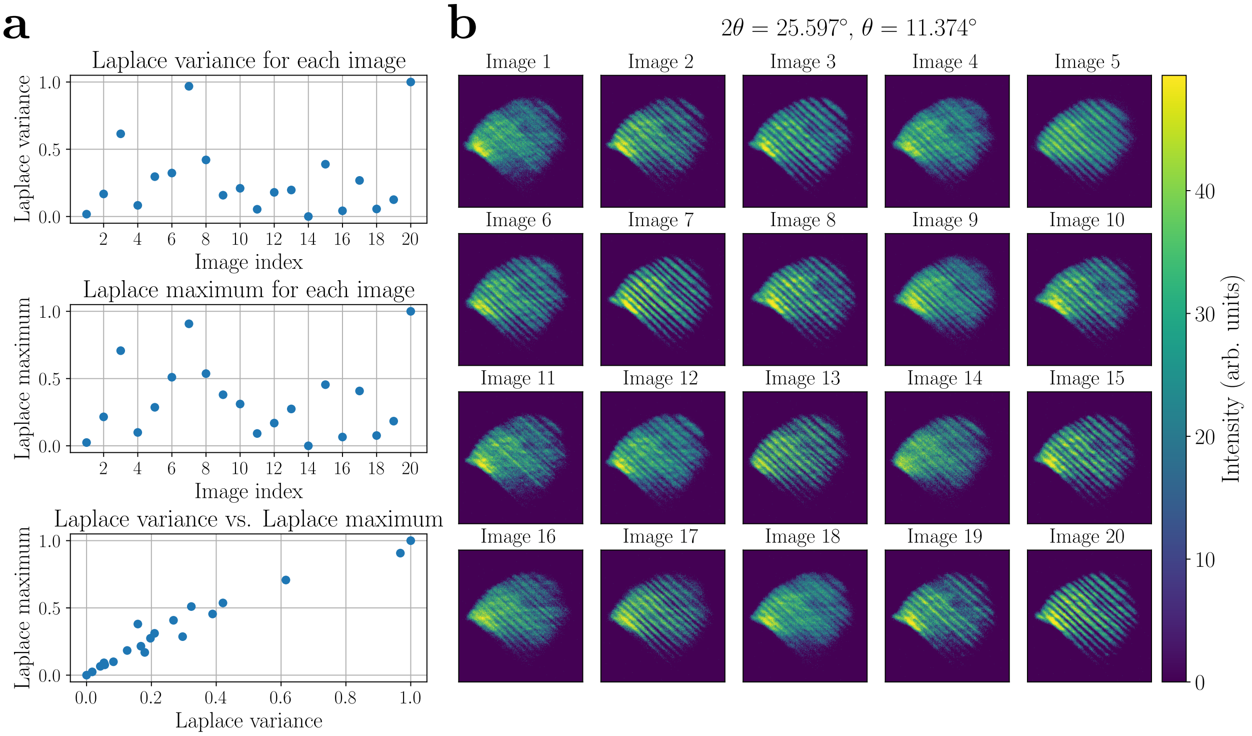

Figure S1: **Automatic detection of the sharpest images from a dataset**

a Laplacian variance and Laplacian maximum distribution for a dataset of 20 images all collected consecutively at the same $(\theta, 2\theta)$ value. The lowest panel shows that these two metrics for quantifying image sharpness track each other almost linearly. **b** The images for which the sharpness metrics are plotted in panel **a**. We can confirm that images determined to be sharp by the Laplacian metrics are indeed visually the sharpest.

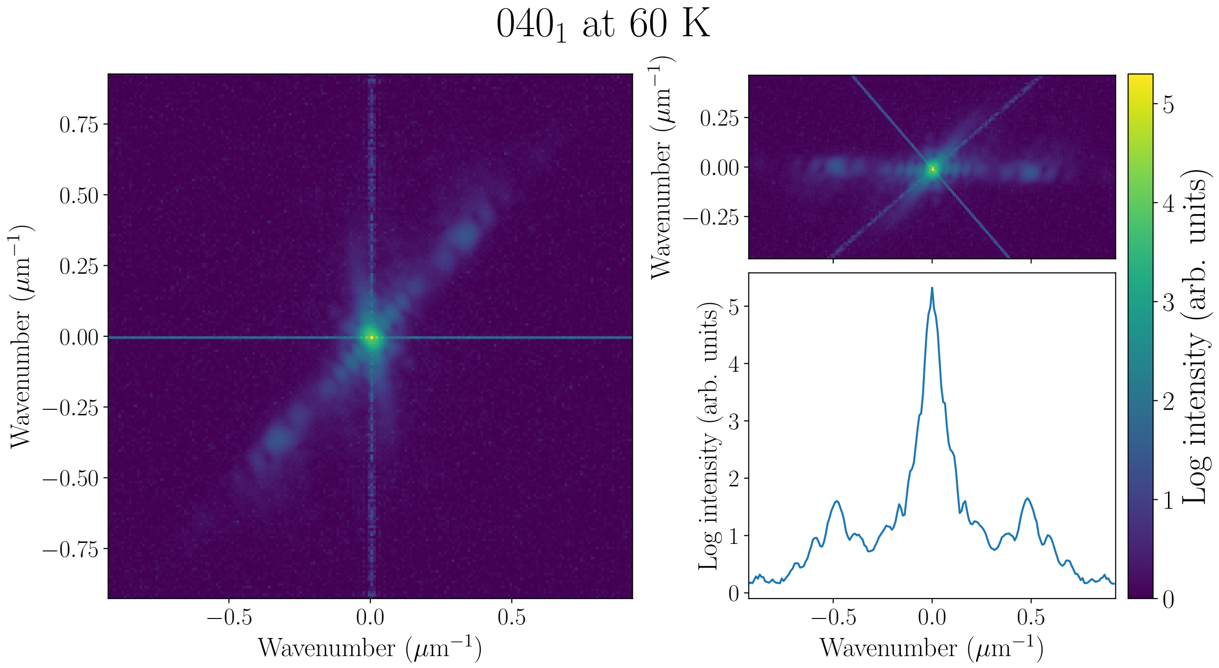

Figure S2: **Wavelength determination from Fourier transform plots**

The aggregate FT plot of the 040_1 peak at 60 K for $2\theta = 25.611°$ from Fig. 4**c** is recreated here. On the right panel, the FT plot rotated as described in the text is shown above and its average taken along the y-axis is shown below. The line plot clearly shows the main satellite peaks at ~0.5 μm^{-1} corresponding to a wavelength of ~2 μm.

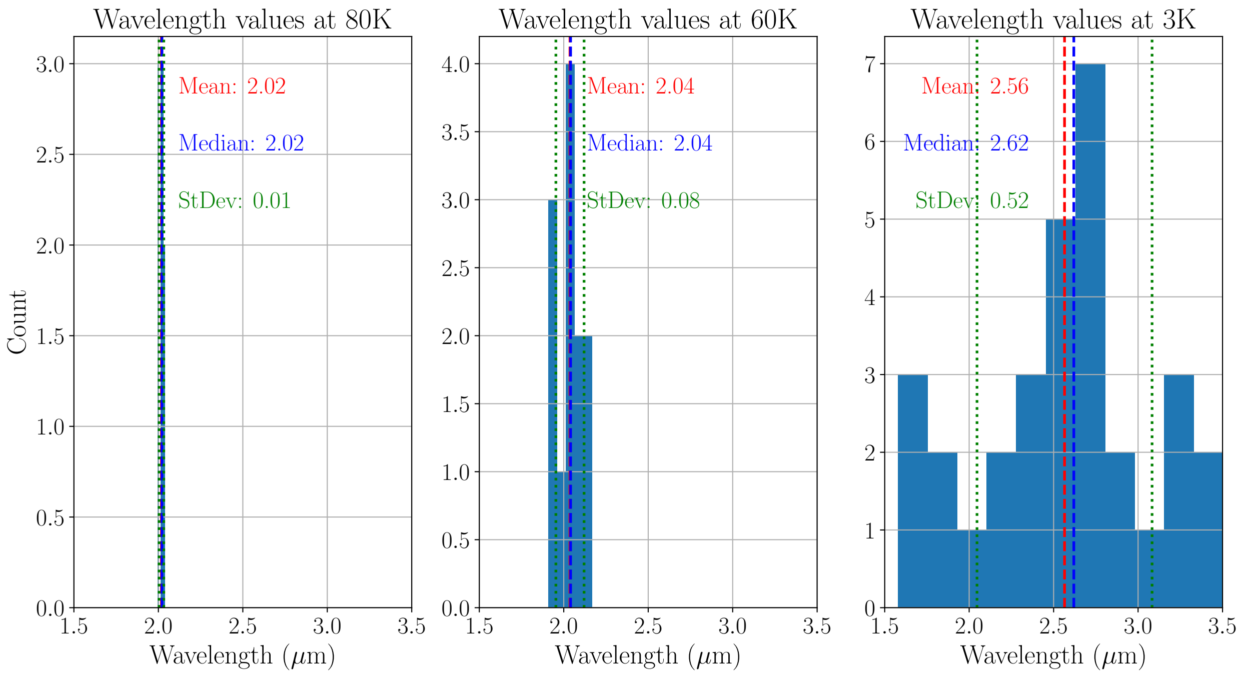

Figure S3: **Histograms of wavelength values determined at different temperatures**

The count of wavelength values determined for a range of 2θ values at 80 K and 60 K, and for range of sample locations at 3 K. The red (blue) dashed lines indicate the mean (median) value of each plot, and the green dotted lines indicate the standard deviation of the sampled data points. The limits of the x-axes for the plots are set to be the same to demonstrate the differing variance of wavelength values detected at each temperature. This difference is discussed in the text.

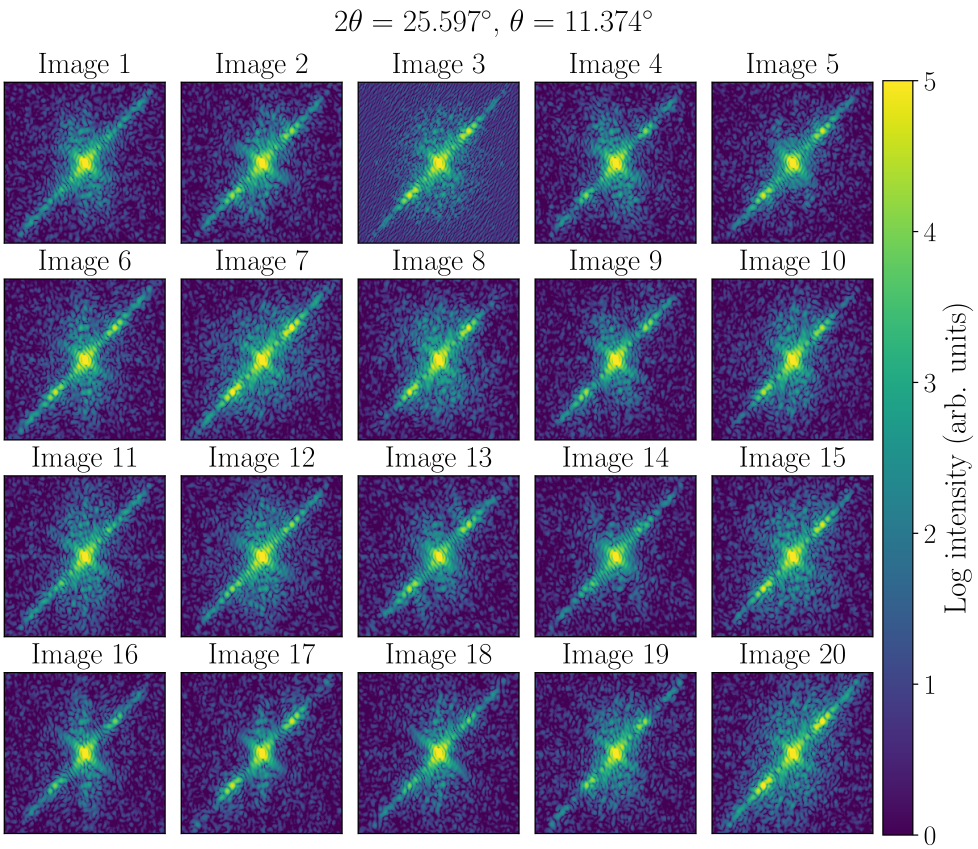

Figure S4: **Fourier transform of individual images from Fig. S1b**

FT plots of individual images also show subdominant peaks along with the dominant satellite peaks and the central peak. The subdominant peaks are unidirectional along the dominant satellite peaks and are also periodic.

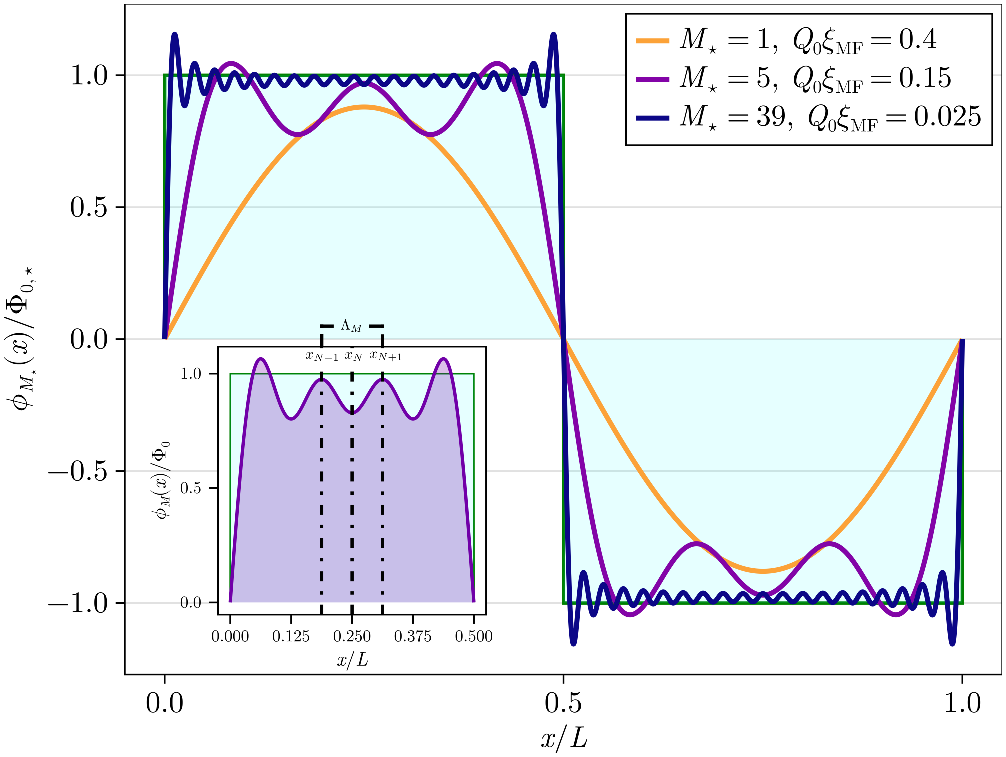

Figure S5: Twin formation through partial square waves as function of temperature. The minimizing twin domain field configuration, $\phi_{M_\star}(x)$, is given for various temperatures, parameterized through the quantity $Q_0\xi_{\mathrm{MF}} \propto 1/\sqrt{T^* - T}$ (see Eq. (S22)). The field configuration is measured relative to $\Phi_{0,\star} = \sqrt{-r/4u}$ – the order parameter associated with uniform long-range order. The perfectly uniform twin domains are shaded for comparison. As the system cools from the nematic critical temperature, T^*, spatial modulations within the bulk of each twin component appear. The length scale is given by Λ_M, as shown in the inset. The vertical lines denote particular extrema, as explained in the text, and it is found that $\Lambda_{M_\star} = L/(M_\star + 1)$.

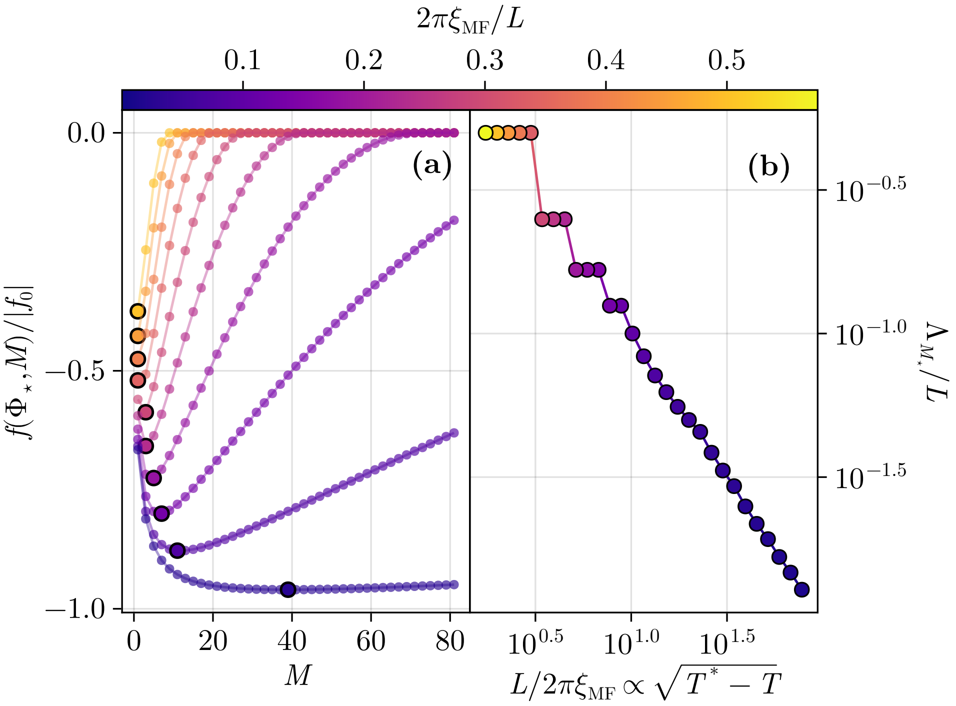

Figure S6: *Ansatz* minimizers as a function within the nematic phase ($T < T^*$). **(a)** The free energy density $f(\Phi_\star, M)$ as a function of the cutoff integer M. The free energy is evaluated at the minimizing amplitude $\Phi_\star$, and is measured relative to the free energy minimum with a uniform nematic order parameter, f_0. The different curves correspond to different temperatures, T, from Eq. (S22). The minimizing cutoff, $M_\star$, is emphasized with a larger outlined point. **(b)** The modulation length within the bulk of a twin component, $\Lambda_{M_\star}$, as a function of the distance from nematic criticality. In both panels, the lines are guides to the eye.

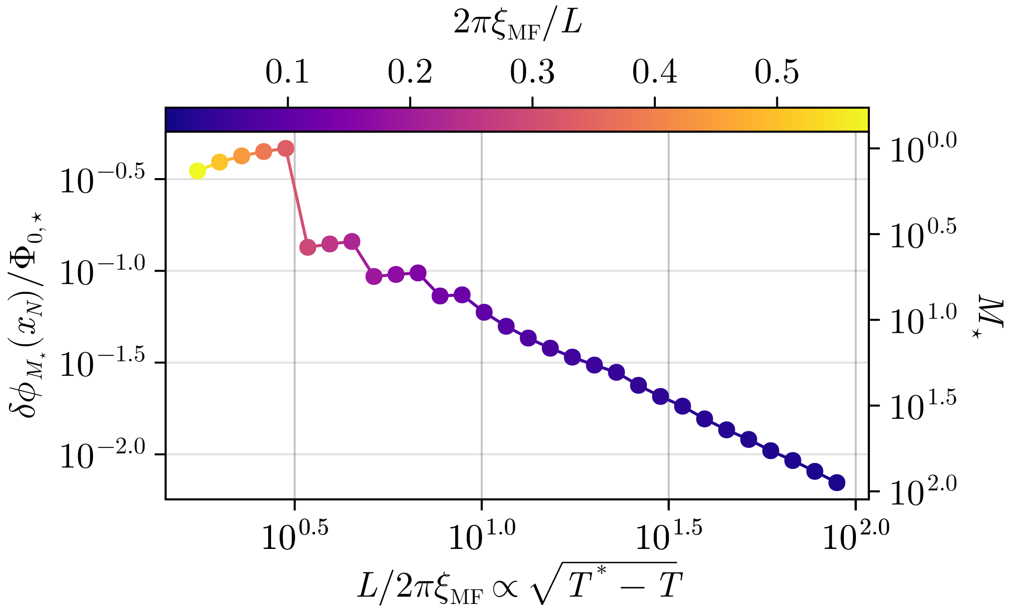

Figure S7: Spatial modulation amplitude within the bulk of the twin domains as a function of temperature. The temperature scale is parameterized through Eq. (S22), and it shows that the bulk modulation amplitude is a slowly decreasing function of temperature deep in the nematic phase. The right descending axis shows the corresponding minimizing cutoff, $M_\star$, in the partial square wave *ansatz*. All axes are on a logarithmic scale. The line is a guide for the eye.

REFERENCES

1. E. K. H. Salje, Ferroelastic materials. *Annu. Rev. Mat. Res.* **42**, 265–283 (2012).

2. A. K. Tagantsev, L. E. Cross, J. Fousek, *Domains in Ferroic Crystals and Thin Films* (Springer, ed. 1, 2010).

3. E. K. H. Salje, *Phase Transitions in Ferroelastic and Co-elastic Crystals* (Cambridge Univ. Press, 1993).

4. S. A. Kivelson, E. Fradkin, V. J. Emery, Electronic liquid-crystal phases of a doped Mott insulator. *Nature* **393**, 550–553 (1998).

5. E. Fradkin, S. A. Kivelson, M. J. Lawler, J. P. Eisenstein, A. P. Mackenzie, Nematic Fermi fluids in condensed matter physics. *Annu. Rev. Condens. Matter Phys.* **1**, 153–178 (2010).

6. R. M. Fernandes, A. V. Chubukov, J. Schmalian, What drives nematic order in iron-based superconductors? *Nat. Phys.* **10**, 97–104 (2014).

7. M. J. Hÿtch, J.-L. Putaux, J.-M. Pénisson, Measurement of the displacement field of dislocations to 0.03 Å by electron microscopy. *Nature* **423**, 270–273 (2003).

8. M. J. Hÿtch, E. Snoeck, R. Kilaas, Quantitative measurement of displacement and strain fields from HREM micrographs. *Ultramicroscopy* **74**, 131–146 (1998).

9. H. Simons, A. B. Haugen, A. C. Jakobsen, S. Schmidt, F. Stöhr, M. Majkut, C. Detlefs, J. E. Daniels, D. Damjanovic, H. F. Poulsen, Long-range symmetry breaking in embedded ferroelectrics. *Nat. Mater.* **17**, 814–819 (2018).

10. J. Chu, J. G. Analytis, K. De Greve, P. L. McMahon, Z. Islam, Y. Yamamoto, I. R. Fisher, In-Plane resistivity anisotropy in an underdoped iron arsenide superconductor. *Science* **329**, 824–826 (2010).

11. J. H. Chu, H. H. Kuo, J. G. Analytis, I. R. Fisher, Divergent nematic susceptibility in an iron arsenide superconductor. *Science* **337**, 710–712 (2012).

12. P. Malinowski, Q. Jiang, J. J. Sanchez, J. Mutch, Z. Liu, P. Went, J. Liu, P. J. Ryan, J.-W. Kim, J.-H. Chu, Suppression of superconductivity by anisotropic strain near a nematic quantum critical point. *Nat. Phys.* **16**, 1189–1193 (2020).

13. A. Steppke, L. Zhao, M. E. Barber, T. Scaffidi, F. Jerzembeck, H. Rosner, A. S. Gibbs, Y. Maeno, S. H. Simon, A. P. Mackenzie, C. W. Hicks, Strong peak in T_c of Sr_2RuO_4 under uniaxial pressure. *Science* **355**, eaaf9398 (2017).

14. J. Straquadine, M. Ikeda, I. Fisher, Evidence for realignment of the charge density wave state in $ErTe_3$ and $TmTe_3$ under uniaxial stress via elastocaloric and elastoresistivity measurements. *Phys. Rev. X* **12**, 021046 (2022).

15. V. Sunko, E. Abarca Morales, I. Marković, M. E. Barber, D. Milosavljević, F. Mazzola, D. A. Sokolov, N. Kikugawa, C. Cacho, P. Dudin, H. Rosner, C. W. Hicks, P. D. C. King, A. P. Mackenzie, Direct observation of a uniaxial stress-driven Lifshitz transition in Sr_2RuO_4. *NPJ Quantum Mater.* **4**, 46 (2019).

16. J. Mutch, W.-C. Chen, P. Went, T. Qian, I. Z. Wilson, A. Andreev, C.-C. Chen, J.-H. Chu, Evidence for a strain-tuned topological phase transition in $ZrTe_5$. *Sci. Adv.* **5**, eaav9771 (2019).

17. H. H. Kuo, J. H. Chu, J. C. Palmstrom, S. A. Kivelson, I. R. Fisher, Ubiquitous signatures of nematic quantum criticality in optimally doped Fe-based superconductors. *Science* **352**, 958–962 (2016).

18. C. W. Hicks, M. E. Barber, S. D. Edkins, D. O. Brodsky, A. P. Mackenzie, Piezoelectric-based apparatus for strain tuning. *Rev. Sci. Instrum.* **85**, 065003 (2014).

19. J. J. Sanchez, P. Malinowski, J. Mutch, J. Liu, J.-W. Kim, P. J. Ryan, J.-H. Chu, The transport-structural correspondence across the nematic phase transition probed by elasto X-ray diffraction. *Nat. Mater.* **20**, 1519–1524 (2021).

20. A. G. Singh, M. D. Bachmann, J. J. Sanchez, A. Pandey, A. Kapitulnik, J. W. Kim, P. J. Ryan, S. A. Kivelson, I. R. Fisher, Emergent tetragonality in a fundamentally orthorhombic material. *Sci. Adv.* **10**, eadk3321 (2024).

21. M. D. Bachmann, G. M. Ferguson, F. Theuss, T. Meng, C. Putzke, T. Helm, K. R. Shirer, Y. S. Li, K. A. Modic, M. Nicklas, M. König, D. Low, S. Ghosh, A. P. Mackenzie, F. Arnold, E. Hassinger, R. D. McDonald, L. E. Winter, E. D. Bauer, F. Ronning, B. J. Ramshaw, K. C. Nowack, P. J. Moll, Spatial control of heavy-fermion superconductivity in $CeIrIn_5$. *Science* **366**, 221–226 (2019).

22. P. J. Moll, Focused ion beam microstructuring of quantum matter. *Annu. Rev. Condens. Matter Phys.* **9**, 147–162 (2018).

23. D. A. Freedman, D. Roundy, T. A. Arias, Elastic effects of vacancies in strontium titanate: Short- and long-range strain fields, elastic dipole tensors, and chemical strain. *Phys. Rev. B* **80**, 064108 (2009).

24. W. W. Schmahl, A. Putnis, E. Saue, P. Freeman, A. Graeme-Barber, R. Jones, K. K. Singh, J. Blunt, P. P. Edwards, J. Loram, K. Mirza, Twin formation and structural modulations in orthorhombic and tetragonal $YBa_2(Cu_{1-x}Co_x)_3O_{7-\delta}$. *Philos. Mag. Lett.* **60**, 241–248 (1989).

25. E. C. Blomberg, A. Kreyssig, M. A. Tanatar, R. M. Fernandes, M. G. Kim, A. Thaler, J. Schmalian, S. L. Bud'ko, P. C. Canfield, A. I. Goldman, R. Prozorov, Effect of tensile stress on the in-plane resistivity anisotropy in $BaFe_2As_2$. *Phys. Rev. B* **85**, 144509 (2012).

26. R. Prozorov, M. A. Tanatar, N. Ni, A. Kreyssig, S. Nandi, S. L. Bud'ko, A. I. Goldman, P. C. Canfield, Intrinsic pinning on structural domains in underdoped single crystals of $Ba(Fe_{1-x}Co_x)_2As_2$. *Phys. Rev. B* **80**, 174517 (2009).

27. H. Simons, A. King, W. Ludwig, C. Detlefs, W. Pantleon, S. Schmidt, F. Stöhr, I. Snigireva, A. Snigirev, H. F. Poulsen, Dark-field X-ray microscopy for multiscale structural characterization. *Nat. Commun.* **6**, 6098 (2015).

28. Z. Qiao, X. Shi, P. Kenesei, A. Last, L. Assoufid, Z. Islam, A large field-of-view high-resolution hard x-ray microscope using polymer optics. *Rev. Sci. Instrum.* **91**, 113703 (2020).

29. L. E. Dresselhaus-Marais, G. Winther, M. Howard, A. Gonzalez, S. R. Breckling, C. Yildirim, P. K. Cook, M. Kutsal, H. Simons, C. Detlefs, J. H. Eggert, H. F. Poulsen, In situ visualization of long-range defect interactions at the edge of melting. *Sci. Adv.* **7**, eabe8311 (2021).

30. J. Plumb, I. Poudyal, R. L. Dally, S. Daly, S. D. Wilson, Z. Islam, Dark field X-ray microscopy below liquid-helium temperature: The case of $NaMnO_2$. *Mater Charact* **204**, 113174 (2023).

31. J. Plumb, A. C. Salinas, K. Mallayya, E. Kisiel, F. B. Carneiro, R. Gomez, G. Pokharel, E.-A. Kim, S. Sarker, Z. Islam, S. Daly, S. D. Wilson, Phase-separated charge order and twinning across length scales in CsV_3Sb_5. *Phys. Rev. Mater.* **8**, 093601 (2024).

32. J.-H. Chu, J. G. Analytis, C. Kucharczyk, I. R. Fisher, Determination of the phase diagram of the electron-doped superconductor $Ba(Fe_{1-x}Co_x)_2As_2$. *Phys. Rev. B* **79**, 014506 (2009).

33. J. Paglione, R. L. Greene, High-temperature superconductivity in iron-based materials. *Nat. Phys.* **6**, 645–658 (2010).

34. R. M. Fernandes, A. I. Coldea, H. Ding, I. R. Fisher, P. J. Hirschfeld, G. Kotliar, Iron pnictides and chalcogenides: A new paradigm for superconductivity. *Nature* **601**, 35–44 (2022).

35. N. Ni, A. Thaler, J. Q. Yan, A. Kracher, E. Colombier, S. L. Bud'ko, P. C. Canfield, S. T. Hannahs, Temperature versus doping phase diagrams for $Ba(Fe_{1-x}TM_x)_2As_2$ (TM = Ni, Cu, Cu/Co) single crystals. *Phys. Rev. B* **82**, 024519 (2010).

36. R. M. Fernandes, M. G. Vavilov, A. V. Chubukov, Enhancement of T_c by disorder in underdoped iron pnictide superconductors. *Phys. Rev. B* **85**, 140512 (2012).

37. H. H. Kuo, M. C. Shapiro, S. C. Riggs, I. R. Fisher, Measurement of the elastoresistivity coefficients of the underdoped iron arsenide $Ba(Fe_{0.975}Co_{0.025})_2As_2$. *Phys. Rev. B* **88**, 085113 (2013).

38. M. A. Tanatar, A. Kreyssig, S. Nandi, N. Ni, S. L. Bud'ko, P. C. Canfield, A. I. Goldman, R. Prozorov, Direct imaging of the structural domains in the iron pnictides AFe_2As_2 (A = Ca, Sr, Ba). *Phys. Rev. B* **79**, 180508 (2009).

39. E. Thewalt, I. M. Hayes, J. P. Hinton, A. Little, S. Patankar, L. Wu, T. Helm, C. V. Stan, N. Tamura, J. G. Analytis, J. Orenstein, Imaging anomalous nematic order and strain in optimally doped $BaFe_2(As,P)_2$. *Phys. Rev. Lett.* **121**, 027001 (2018).

40. F. Yang, S. F. Taylor, S. D. Edkins, J. C. Palmstrom, I. R. Fisher, B. L. Lev, Nematic transitions in iron pnictide superconductors imaged with a quantum gas. *Nat. Phys.* **16**, 514–519 (2020).

41. B. Kalisky, J. R. Kirtley, J. G. Analytis, J.-H. Chu, A. Vailionis, I. R. Fisher, K. A. Moler, Stripes of increased diamagnetic susceptibility in underdoped superconducting $Ba(Fe_{1-x}Co_x)_2As_2$ single crystals: Evidence for an enhanced superfluid density at twin boundaries. *Phys. Rev. B* **81**, 184513 (2010).

42. L. Stojchevska, T. Mertelj, J.-H. Chu, I. R. Fisher, D. Mihailovic, Doping dependence of femtosecond quasiparticle relaxation dynamics in $Ba(Fe,Co)_2As_2$ single crystals: Evidence for normal-state nematic fluctuations. *Phys. Rev. B* **86**, 024519 (2012).

43. M. A. Tanatar, E. C. Blomberg, A. Kreyssig, M. G. Kim, N. Ni, A. Thaler, S. L. Bud'ko, P. C. Canfield, A. I. Goldman, I. I. Mazin, R. Prozorov, Uniaxial-strain mechanical detwinning of $CaFe_2As_2$ and $BaFe_2As_2$ crystals: Optical and transport study. *Phys. Rev. B* **81**, 184508 (2010).

44. H. Simons, A. C. Jakobsen, S. R. Ahl, C. Detlefs, H. F. Poulsen, Multiscale 3D characterization with dark-field x-ray microscopy. *MRS Bull.* **41**, 454–459 (2016).

45. C. Yildirim, P. Cook, C. Detlefs, H. Simons, H. F. Poulsen, Probing nanoscale structure and strain by dark-field x-ray microscopy. *MRS Bull.* **45**, 277–282 (2020).

46. H. F. Poulsen, A. C. Jakobsen, H. Simons, S. R. Ahl, P. K. Cook, C. Detlefs, X-ray diffraction microscopy based on refractive optics. *J. Appl. Cryst.* **50**, 1441–1456 (2017).

47. E. Kisiel, P. Salev, I. Poudyal, D. J. Alspaugh, F. Carneiro, E. Qiu, F. Rodolakis, Z. Zhang, O. G. Shpyrko, M. Rozenberg, I. K. Schuller, Z. Islam, A. Frano, High-resolution full-field structural microscopy of the voltage-induced filament formation in VO_2-based neuromorphic devices. *ACS Nano* **19**, 15385–15394 (2025).

48. E. Kisiel, I. Poudyal, P. Kenesei, M. Engbretson, A. Last, R. Basak, I. Zaluzhnyy, U. Goteti, R. Dynes, A. Miceli, A. Frano, Z. Islam, Direct detection system for full-field nanoscale X-ray diffraction-contrast imaging. *Opt. Express* **32**, 27682–27689 (2024).

49. H. Kleinert, "Part III: Gauge fields in solids," in *Gauge Fields in Condensed Matter* (World Scientific, 1989), vol. 2, pp. 745–1329.

50. W. J. Meese, R. M. Fernandes, Compatible instability: Gauge constraints of elasticity inherited by electronic nematic criticality. arXiv:2507.23753 [cond-mat.str-el] (2025).

51. W. J. Meese, R. M. Fernandes, Theory of electronic nematic criticality constrained by elastic compatibility. arXiv:2507.23754 [cond-mat.str-el] (2025).

52. K. O. Rasmussen, T. Lookman, A. Saxena, A. R. Bishop, R. C. Albers, S. R. Shenoy, Three-dimensional elastic compatibility and varieties of twins in martensites. *Phys. Rev. Lett.* **87**, 055704 (2001).

53. R. T. Brierley, P. B. Littlewood, Domain wall fluctuations in ferroelectrics coupled to strain. *Phys. Rev. B* **89**, 184104 (2014).

54. I. Paul, M. Garst, Lattice effects on nematic quantum criticality in metals. *Phys. Rev. Lett.* **118**, 227601 (2017).

55. E. Dagotto, Complexity in strongly correlated electronic systems. *Science* **309**, 257–262 (2005).

56. K. Lai, M. Nakamura, W. Kundhikanjana, M. Kawasaki, Y. Tokura, M. A. Kelly, Z. X. Shen, Mesoscopic percolating resistance network in a strained manganite thin film. *Science* **329**, 190–193 (2010).

57. Z. Jin, S. Ismail-Beigi, First-principles prediction of structural distortions in the cuprates and their impact on the electronic structure. *Phys. Rev. X* **14**, 041053 (2024).

58. D. Gürsoy, K. A. Yay, E. Kisiel, M. Wojcik, D. Sheyfer, A. Last, M. Highland, I. R. Fisher, S. Hruszkewycz, Z. Islam, Dark-field X-ray microscopy with structured illumination for three-dimensional imaging. *Commun. Phys.* **8**, 34 (2025).

59. A. Lahiri, A. Klein, R. M. Fernandes, Defect-induced electronic smectic state at the surface of nematic materials. *Phys. Rev. B* **106**, L140503 (2022).

60. M. S. Ikeda, T. Worasaran, E. W. Rosenberg, J. C. Palmstrom, S. A. Kivelson, I. R. Fisher, Elastocaloric signature of nematic fluctuations. *Proc. Natl. Acad. Sci. U.S.A.* **118**, e2105911118 (2021).

61. S. Lederer, Y. Schattner, E. Berg, S. A. Kivelson, Enhancement of superconductivity near a nematic quantum critical point. *Phys. Rev. Lett.* **114**, 097001 (2015).

62. A. T. Hristov, M. S. Ikeda, J. C. Palmstrom, P. Walmsley, I. R. Fisher, Elastoresistive and elastocaloric anomalies at magnetic and electronic-nematic critical points. *Phys. Rev. B* **99**, 100101 (2019).

63. J. Garriga Ferrer, R. Rodríguez-Lamas, H. Payno, W. De Nolf, P. Cook, V. A. Solé Jover, C. Yildirim, C. Detlefs, *darfix* – Data analysis for dark-field X-ray microscopy. *J. Synchrotron Radiat.* **30**, 527–537 (2023).

64. R. Bansal, G. Raj, T. Choudhury, "Blur image detection using Laplacian operator and Open-CV," in *2016 International Conference System Modeling & Advancement in Research Trends (SMART)* (IEEE, 2017), pp. 63–67.

65. S. Pertuz, D. Puig, M. A. Garcia, Analysis of focus measure operators for shape-from-focus. *Pattern Recognit.* **46**, 1415–1432 (2013).

66. R. M. Fernandes, A. V. Chubukov, J. Knolle, I. Eremin, J. Schmalian, Preemptive nematic order, pseudogap, and orbital order in the iron pnictides. *Phys. Rev. B* **85**, 024534 (2012).

67. W. J. Meese, "Consequences of nematoelasticity in structurally disordered quantum materials," thesis, University of Minnesota Twin Cities (2024).

68. J. D. Eshelby, "The Continuum Theory of Lattice Defects," in *Solid State Physics*, F. Seitz, D. Turnbull, Eds. (Academic Press, 1956), vol. 3, pp. 79–144.

69. R. deWit, "Linear theory of static dislocations," in *Fundamental Aspects of Dislocation Theory*, J. A. Simmons, R. deWit, R. Bullough, Eds. (National Bureau of Standards, 1970), vol. 1, pp. 651–673.

70. R. deWit, Theory of disclinations: II. Continuous and discrete disclinations in anisotropic elasticity. *J. Res. Natl. Bur. Stand. A Phys. Chem.* **77A**, 49–100 (1973).

71. R. deWit, Theory of disclinations: III. Continuous and discrete disclinations in isotropic elasticity. *J. Res. Natl. Bur. Stand. A Phys. Chem.* **77A**, 359–368 (1973).

72. R. deWit, Theory of disclinations: IV. Straight disclinations. *J. Res. Natl. Bur. Stand. A Phys. Chem.* **77A**, 607–658 (1973).

73. E. Kröner, "Continuum theory of defects," in *Physics of Defects*, Les Houches, Session 35, R. Balian, M. Kléman, J.-P. Poirier, Eds. (North-Holland Pub. Co., Amsterdam, 1981), pp. 215–315.

74. T. Mura, *Micromechanics of Defects in Solids*, vol. 3 of *Mechanics of Elastic and Inelastic Solids* (Springer, ed. 2, 1987).

75. R. Gröger, T. Lookman, A. Saxena, Defect-induced incompatibility of elastic strains: Dislocations within the Landau theory of martensitic phase transformations. *Phys. Rev. B* **78**, 184101 (2008).

76. A. J. Beekman, J. Nissinen, K. Wu, K. Liu, R.-J. Slager, Z. Nussinov, V. Cvetkovic, J. Zaanen, Dual gauge field theory of quantum liquid crystals in two dimensions. *Phys. Rep.* **683**, 1–110 (2017).

77. M. Pretko, L. Radzihovsky, Fracton-elasticity duality. *Phys. Rev. Lett.* **120**, 195301 (2018).

78. M. Pretko, Z. Zhai, L. Radzihovsky, Crystal-to-fracton tensor gauge theory dualities. *Phys. Rev. B* **100**, 134113 (2019).

79. J. Gaa, G. Palle, R. M. Fernandes, J. Schmalian, Fracton-elasticity duality in twisted moiré superlattices. *Phys. Rev. B* **104**, 064109 (2021).

80. R. A. Cowley, Acoustic phonon instabilities and structural phase transitions. *Phys. Rev. B* **13**, 4877–4885 (1976).

81. R. Folk, H. Iro, F. Schwabl, Critical statics of elastic phase transitions. *Z. Phys. B: Condens. Matter* **25**, 69–81 (1976).

82. U. Karahasanovic, J. Schmalian, Elastic coupling and spin-driven nematicity in iron-based superconductors. *Phys. Rev. B* **93**, 064520 (2016).

83. R. M. Fernandes, J. W. F. Venderbos, Nematicity with a twist: Rotational symmetry breaking in a moiré superlattice. *Sci. Adv.* **6**, eaba8834 (2020).

84. M. Hecker, R. M. Fernandes, Phonon-induced rotation of the electronic nematic director in superconducting Bi_2Se_3. *Phys. Rev. B* **105**, 174504 (2022).

85. L. D. Landau, E. M. Lifshitz, *Theory of Elasticity*, vol. 7 of *Course of Theoretical Physics* (Pergamon Press, ed. 2, 1970).
